# A Q-Learning-Based Hyper-Heuristic Genetic Algorithm for Optimizing Human–Robot Collaborative Assembly Lines

**DOI:** 10.3390/biomimetics11080600

**Published:** 2026-08-21

**Authors:** Seçil Kulaç

**Affiliations:** Quality Coordination Office, Bursa Technical University, 16350 Bursa, Türkiye; secil.kulac@btu.edu.tr

**Keywords:** assembly line balancing and scheduling, bio-inspired optimization, energy expenditure, genetic algorithm, human–robot collaboration, hyper-heuristic, Q-learning

## Abstract

Human–robot collaborative assembly line balancing and scheduling constitutes an NP-hard combinatorial optimization problem involving the simultaneous optimization of task assignment, resource allocation, processing mode selection, station-level scheduling, and ergonomic constraints. This study proposes a Q-learning-based hyper-heuristic genetic algorithm (QLHH-GA) to solve the cost-oriented ergonomic mixed-model human–robot collaborative assembly line balancing and scheduling problem. The proposed approach integrates bio-inspired evolutionary mechanisms of population variation and selection with adaptive, Q-learning-guided low-level heuristic selection. The Q-learning layer uses performance feedback to adapt the search strategy to different solution states while maintaining solution feasibility. A mixed-integer linear programming (MILP) model is also developed to minimize the total operating cost, including station opening, labor, robot operation, and energy consumption costs, while enforcing station-level energy expenditure (EE) limits. Computational experiments conducted using benchmark instances of varying sizes and a literature-based industrial case study demonstrate that QLHH-GA produces solutions comparable to those obtained by the MILP model on small-scale instances and maintains strong solution quality on larger instances, for which exact optimization becomes computationally prohibitive. These findings demonstrate the scalability and effectiveness of reinforcement-learning-guided hyper-heuristic search for designing cost-efficient and ergonomically constrained human–robot collaborative assembly lines.

## 1. Introduction

Assembly line systems remain a central component of modern manufacturing, supporting high-volume production while maintaining operational consistency. With the adoption of digital technologies under the Industry 4.0 paradigm, manufacturing systems have evolved to exhibit increased automation, connectivity, and data-driven decision-making. Within this transformation, human–robot collaboration (HRC) has emerged as an approach that combines human adaptability and cognitive capabilities with the precision and repeatability of robotic systems [1].

The transition to Industry 5.0 introduces a complementary perspective that emphasizes human-centric, sustainable production systems. In contrast to earlier paradigms that focused mainly on efficiency and automation, Industry 5.0 highlights the importance of integrating advanced technologies in a way that supports worker well-being and system resilience [2]. In this context, collaborative assembly systems have attracted increasing attention as they enable humans and robots to operate in shared environments while addressing both operational and human-related objectives.

Despite these developments, assembly line balancing remains a complex optimization problem. Straight assembly lines, widely used in industrial practice due to their structural simplicity and ease of implementation, require careful coordination of tasks and resources. The assembly line balancing problem (ALBP), first introduced by Salveson [3], involves assigning tasks to workstations under precedence constraints while optimizing specific performance metrics. In HRC environments, this problem becomes more complex due to the presence of heterogeneous resources, alternative processing modes, and interaction requirements between operators [1].

Recent studies have increasingly emphasized integrating scheduling decisions into assembly line models. In HRC systems, tasks can be executed sequentially, in parallel, or collaboratively, which introduces additional constraints on synchronization and feasibility. Therefore, the joint consideration of task assignment, resource allocation, and scheduling decisions is necessary to represent real-world production settings more accurately.

Ergonomic risk is an important consideration in assembly line design. Repetitive tasks, awkward postures, and physically demanding operations may lead to work-related musculoskeletal disorders (WMSDs), which can negatively affect both worker health and system performance. Therefore, integrating ergonomic considerations into optimization models has received increasing attention. Among the available approaches, energy expenditure (EE) is frequently adopted because it provides a continuous and analytically tractable measure of physical workload that can be incorporated into mathematical formulations [4]. Introducing station-level limits on energy expenditure allows ergonomic risks to be controlled within the decision-making process.

From a methodological perspective, the increasing complexity of HRC assembly line balancing problems has substantially reduced the applicability of exact optimization methods, particularly for medium- and large-scale instances. Consequently, nature-inspired optimization algorithms, including evolutionary algorithms and swarm intelligence methods, have been extensively employed because of their ability to efficiently explore complex search spaces within acceptable computational times. Nevertheless, most existing metaheuristic approaches rely on predefined search operators and fixed search strategies, which may limit their adaptability to different problem characteristics. To improve search adaptability, recent studies have increasingly investigated hybrid optimization frameworks that integrate reinforcement learning and hyper-heuristic mechanisms with evolutionary algorithms, enabling adaptive operator selection based on the current search state. Such hybrid frameworks provide a more flexible search process by dynamically selecting search operators according to the characteristics of the current solution, thereby improving the balance between exploration and exploitation.

Recent research has shifted toward adaptive and learning-based optimization methods. In particular, the integration of reinforcement learning with metaheuristic algorithms has attracted increasing attention. Q-learning enables adaptive decision-making by learning from interactions with the environment and evaluating actions based on long-term rewards [5]. When combined with metaheuristic frameworks, it supports dynamic operator selection and improves the balance between exploration and exploitation of the solution space.

Hyper-heuristic approaches provide a higher-level strategy by managing a set of low-level heuristics and adaptively selecting them during the search process. The integration of Q-learning and hyper-heuristic mechanisms improves search efficiency and solution robustness in complex optimization problems [5]. However, studies that simultaneously incorporate ergonomic constraints, scheduling decisions, and adaptive learning-based solution approaches in straight assembly line configurations remain limited.

In this context, this study investigates an ergonomic mixed-model assembly line balancing problem in a straight-line configuration under human–robot collaboration. A mixed-integer linear programming (MILP) model is developed to minimize the total cost while incorporating ergonomic constraints through energy expenditure limits. Scheduling considerations are also included to ensure feasibility for different execution modes. A Q-learning-based hyper-heuristic genetic algorithm (QLHH-GA) is proposed to solve large-scale instances by adaptively selecting neighborhood structures based on their performance.

The main contributions of this study are summarized as follows:

- A comprehensive MILP formulation is developed for the straight-line mixed-model human–robot collaborative assembly line balancing and scheduling problem, jointly considering task assignment, resource allocation, processing mode selection, and scheduling decisions within a cost minimization framework.
- A bio-inspired evolutionary optimization framework is developed by combining the population-based variation and selection mechanisms of a genetic algorithm with Q-learning-guided low-level heuristic selection.
- Ergonomic risk is explicitly integrated into the optimization model through energy expenditure (EE), enabling continuous workload evaluation and the enforcement of station-level ergonomic constraints.
- An adaptive reinforcement learning mechanism is developed to dynamically select low-level heuristics according to the current search state, improving search adaptability and maintaining an effective balance between exploration and exploitation.

The remainder of this paper is organized as follows. Section 2 reviews the relevant literature. Section 3 presents the problem description and proposed mathematical formulations. Section 4 introduces the solution methodology, including the Q-learning-based hyper-heuristic genetic algorithm. Section 5 reports the computational experiments and discusses the results. Finally, Section 6 concludes the study and outlines directions for future research.

## 2. Literature Review

The ALBPs have been widely studied, and their theoretical foundations and classifications have been reviewed by Becker and Scholl [6] and Battaia and Dolgui [7]. The earliest mathematical formulation of the ALBP dates back to Salveson [3], and subsequent studies have expanded the problem by incorporating exact, heuristic, and meta-heuristic solution approaches [8]. With the transition toward Industry 4.0 and 5.0 paradigms, research has increasingly focused on HRC assembly systems, where productivity improvements are pursued alongside human-centered considerations such as ergonomics, safety, and sustainability. Within this line of work, Kheirabadi et al. [9] and Chutima [1] indicate that despite significant progress, the integration of ergonomic factors and realistic operational constraints into assembly line balancing models remains limited.

At the same time, production environments have evolved from single-line to multi-line and mixed-model systems, driven by the need for flexibility and efficiency. Multiple assembly line systems allow the simultaneous production of different product models; however, many studies assume predefined model assignments, limiting their applicability in dynamic production settings. In particular, the joint optimization of model-line assignment and assembly line balancing has received limited attention, despite its significant impact on system performance. Moreover, the integration of collaborative cobots further increases the complexity of these systems owing to heterogeneous resource capabilities, energy consumption, and synchronization requirements.

In addition to traditional balancing decisions, recent research has increasingly emphasized the integration of scheduling into assembly line designs. In HRC systems, tasks may be executed sequentially, in parallel, or collaboratively, which increases the complexity of ensuring feasible schedules. Nourmohammadi et al. [10] demonstrated that the integrated optimization of task assignment, resource allocation, and scheduling leads to more realistic and efficient solutions. Similarly, Vieira et al. [11] proposed an optimization–simulation framework for planning and scheduling HRC systems. Shan et al. [12] developed a multi-objective model for HRC assembly line balancing by jointly considering assembly mode selection, task allocation, and sequencing decisions, whereas Huang et al. [13] considered parallel task execution and coordination between human and robotic operations. The latter study also highlighted the importance of synchronization in ensuring feasibility under collaborative execution modes. Overall, these findings indicate that scheduling should be integrated into assembly line balancing rather than treated as a separate decision layer.

Building on these developments, a considerable portion of the literature has focused on cost-oriented formulations of HRC assembly line balancing. In these studies, the primary objective is typically defined as minimizing the total operational cost, including station opening, labor, and robot-related costs. Weckenborg and Spengler [14] proposed a MILP model that incorporates ergonomic load into cost-based decision-making. Dalle Mura and Dini [15] extended this framework by considering worker skill levels and workload distribution and solved the problem using a genetic algorithm (GA). Yaphiar et al. [16] addressed mixed-model production environments and incorporated machinery-related costs into a MILP formulation. Li et al. [17] proposed a multi-objective model minimizing both cost and cycle time and employed a migrating bird optimization approach. Kökhan and Baykoç [18] integrated cost and system configuration decisions and solved the problem using simulated annealing (SA).

More recent studies have extended cost formulations by incorporating energy consumption and environmental considerations in human–robot collaborative assembly systems. In this context, Yilmaz et al. [19] developed a multi-objective model integrating model-line assignment and multi-line balancing with cost, energy consumption, and workload considerations. Subsequently, Yilmaz et al. [20] extended this framework by incorporating environmental impact through PM2.5 emissions arising from cobot energy consumption and solving the problem using NSGA-II. These studies show that integrated decision-making improves both operational efficiency and environmental performance. Kang and Lee [21] addressed a multi-objective mixed-model HRC assembly line balancing problem by jointly minimizing cycle time, collaboration cost, smoothness index, energy consumption, and the number of stations using preemptive fuzzy programming and NSGA-II.

Concurrently, increasing attention has been directed toward incorporating ergonomic considerations into assembly line design. In HRC environments, ergonomic risk is a critical performance indicator owing to the direct interaction between human operators and robotic systems. Among the various assessment methods, EE has been widely adopted because it provides a continuous and analytically tractable representation of the physical workload that can be directly embedded into optimization models [22]. Stecke and Mokhtarzadeh [23] developed a mathematical programming model that integrates ergonomic risk with operational decisions, whereas Keshvarparast et al. [24] addressed human workload minimization within an optimization framework. Bekdemir and Taşan [25] incorporated EE-based constraints into MILP modeling. Cai et al. [26] proposed a task allocation model for human–robot collaborative assembly lines that jointly considers automation potential, assembly complexity, and worker workload. The model was solved using an enhanced multi-objective migratory bird optimization algorithm with fast non-dominated sorting, and the results demonstrated its effectiveness in reducing complexity while improving workload balance without compromising efficiency. More recently, Keshvarparast et al. [27] developed an integrated Learning–Forgetting–Fatigue–Recovery framework for HRC systems, where energy expenditure is used to model physical fatigue and rest allowance requirements within a dynamic optimization structure. Their study highlights that fatigue, recovery, and human performance dynamics should be jointly considered, as neglecting these interactions may lead to inaccurate estimation of production time and worker capability. Kulaç and Kiraz [28] developed a mixed-integer linear programming model for mixed-model human–robot collaborative assembly lines, where multi-dimensional ergonomic factors, including physical, environmental, and psychosocial risks, were explicitly incorporated into the balancing process. The findings demonstrate that integrating comprehensive ergonomic considerations can significantly reduce ergonomic risks while maintaining or improving production efficiency.

From a methodological perspective, nature-inspired optimization algorithms have been widely employed for assembly line balancing problems, particularly when exact methods become computationally impractical. More recently, adaptive and learning-based mechanisms have been integrated into these algorithms to improve search adaptability and solution quality. Deliktaş and Aydin [29] proposed an artificial bee colony-based hyper-heuristic algorithm for the simple assembly line balancing problem, demonstrating improved solution quality through adaptive search mechanisms and local search integration. Similarly, Özbakır and Seçme [30] developed a hyper-heuristic approach for stochastic and parallel assembly line balancing problems, enabling effective exploration of heuristic search spaces while accounting for additional constraints such as equipment costs and uncertainty. In addition to classical balancing problems, Karas Celik and Ozcelik [2] proposed a hyper-matheuristic framework that combines artificial bee colony algorithms with mathematical programming for assembly line rebalancing problems under human–robot collaboration, highlighting the effectiveness of hybrid strategies in handling complex decision structures involving resource allocation and system reconfiguration. In recent studies, reinforcement learning has emerged as a promising approach for assembly line balancing. In this context, Rauf et al. [31] proposed a Q-learning-based hybrid genetic algorithm for mixed-model assembly lines. The method simultaneously addresses balancing and sequencing decisions within a multi-objective optimization framework. Their approach integrates genetic algorithms with reinforcement learning-based parameter tuning, enabling adaptive control of search parameters and improved convergence performance. Subsequently, Baykasoğlu et al. [32] introduced a reinforcement learning-based method for assembly line balancing problems, demonstrating its effectiveness in learning decision policies through interaction with the environment and achieving competitive performance compared with classical heuristics and metaheuristics. Despite these advances, the integration of reinforcement learning with metaheuristic and hyper-heuristic frameworks remains limited, particularly in problems involving ergonomic constraints, energy considerations, and integrated scheduling decisions.

To provide a structured overview of the existing studies, Table 1 summarizes the representative solution approaches in the literature regarding cost-oriented objectives and ergonomic considerations. The table highlights the diversity of the modeling frameworks, ergonomic assessment methods, and solution techniques adopted in HRC assembly line balancing problems.

The reviewed literature reveals several research gaps. First, although cost minimization has been extensively studied, ergonomic and environmental considerations are often treated as secondary objectives rather than enforced as explicit constraints within the optimization framework. Second, although scheduling decisions and energy-related factors have received increasing attention, their integration with ergonomic constraints within a unified model remains limited in the literature. Third, although metaheuristic approaches are widely applied, adaptive hybrid methods that combine reinforcement learning with hyper-heuristic strategies remain underexplored, particularly in mixed-model human–robot collaborative assembly lines. These findings highlight the need for integrated modeling and solution approaches that jointly address cost, ergonomics, and scheduling within adaptive optimization frameworks.

## 3. Problem Description

This study considers a straight assembly line with a unidirectional task flow, where tasks are assigned sequentially to stations while satisfying precedence constraints. The problem is defined as the straight ergonomic mixed-model assembly line balancing problem with human–robot collaboration (Ergo-MMALBP-HRC).

Three processing alternatives are defined for each task: human execution, robot execution, and human–robot collaborative execution, each associated with different processing times and ergonomic impacts.

The problem simultaneously determines: (i) task-to-station assignment, (ii) allocation of human operators and robots to stations, (iii) selection of processing alternatives, and (iv) scheduling of tasks within stations. Precedence relations are enforced across stations in a forward direction, while station-level scheduling ensures feasibility with respect to resource conflicts and intra-station precedence.

The objective is to minimize the total cost per production cycle, including station opening costs, human labor costs, robot operating costs, and energy consumption costs associated with robot-assisted operations. Robot energy consumption is estimated based on processing times, energy consumption rates, and unit energy costs, reflecting the trade-off between fixed and variable operational costs.

The model is based on the following assumptions:

- Task precedence relations, ergonomic risk values, and processing times are known.
- Human operators are homogeneous, whereas robots may differ in capabilities.
- A capability matrix defines feasible task–resource assignments.
- Positive zoning constraints require certain tasks to be assigned to the same station, whereas negative zoning constraints prevent specific tasks from being assigned to the same station.
- Mixed-model production is considered.
- The cost structure includes station opening, labor, robot operation, and energy consumption costs.
- Each operator is assigned to exactly one station, and each station accommodates at most one robot.
- Setup, loading, and unloading times are neglected.
- Operator travel times are not considered.
- No physical interference occurs between adjacent stations.
- Within each station, tasks are processed according to resource availability and scheduling constraints.

Human operators are assumed to have comparable skill levels. In practice, skill differences may affect task eligibility and processing times. Such differences can be incorporated through worker-specific processing times and task–worker compatibility parameters without changing the general structure of the optimization framework. Additionally, setup, loading, unloading, and operator travel times are neglected because the model considers a fixed-station assembly environment in which these times are relatively small compared with task processing times. When significant, they can be incorporated as additional or sequence-dependent processing times.

### 3.1. Ergonomic Risk Assessment

Ergonomic risk in assembly systems is mainly linked to physical workload caused by repetitive motions, sustained postures, and task intensity. This exposure can contribute to work-related musculoskeletal disorders [42]. In optimization-based assembly line design, representing ergonomic risk in a quantitative and model-compatible form is essential.

In this study, ergonomic risk is quantified using energy expenditure (EE), which represents the metabolic energy required to perform a task. EE is adopted because it provides a continuous and analytically tractable measure that can be directly incorporated into mathematical formulations [13,23,43]. For each task, the total energy expenditure consists of the energy required to maintain working postures and to perform elementary actions and is calculated as follows:

(1) $$EE=\sum_{b=1}^{B}P_{b}t_{b}+\sum_{a=1}^{A}M_{a}d_{a}$$

where B is the number of body postures, A is the number of elementary actions, $P_{b}$ is the metabolic energy rate required to maintain posture b (cal/min), $t_{b}$ is the duration of posture b (min), $M_{a}$ is the metabolic energy rate required to perform elementary action a (cal/min), and $d_{a}$ is the duration of action a (min) [13,23].

Cai et al. [26] classify energy expenditure into six workload categories: very light work (<2.5 kcal/min), light work (2.51–4.99 kcal/min), moderate work (5.0–7.5 kcal/min), heavy work (7.51–10.0 kcal/min), very heavy work (10.01–12.49 kcal/min), and extremely heavy work (>12.5 kcal/min). This classification supports the interpretation of *EE* values within established workload levels.

### 3.2. Mathematical Model

This section introduces the mathematical formulation of the Straight Ergo-MMALBP-HRC, which extends the classical assembly line balancing problem by incorporating HRC and ergonomic risk considerations. The proposed model is developed by adapting and extending existing formulations in the literature, particularly those presented by Nourmohammadi et al. [10], Weckenborg et al. [39], Yilmaz et al. [20], and Kulaç and Kiraz [28]. A comprehensive list of notations used in the model is provided in Table 2.

Objective function:

(2) $$Minimize\;z=c\sum_{k=1}^{NS}{cs}_{k}{ws}_{k}+c\sum_{k=1}^{NS}{ch}_{k}h_{k}+\;c\sum_{k=1}^{NS}\sum_{r=1}^{NR}{cr}_{r}z_{rk}+\;\sum_{k=1}^{NS}\sum_{r=1}^{NR}\sum_{i=1}^{NT}\sum_{m=1}^{NM}\sum_{p\in \left\{\sigma ,\tau \right\}}{{ce}_{r}t_{imp}(x}_{impk})$$

Constraints:

(3) $$\sum_{k=1}^{NS}\sum_{p\in \left\{h,\sigma ,\tau \right\}}x_{impk}=1\;\forall i\in \left\{1,2,\ldots ,NT\right\},\;\forall m\in \left\{1,2,\ldots ,NM\right\}$$

(4) $$\sum_{p\;\in \left\{\sigma ,\tau \right\}}x_{impk}\leq \sum_{r=1}^{NR}z_{rk}\;\forall i\in \left\{1,2,\ldots ,NT\right\},\;\forall k\in \left\{1,2,\ldots ,NS\right\},\;\forall m\in \left\{1,2,\ldots ,NM\right\}$$

(5) $$\sum_{k=1}^{NS}z_{rk}\leq 1\;\forall r\in \left\{1,2,\ldots ,NR\right\}$$

(6) $$\sum_{r=1}^{NR}z_{rk}\leq 1\;\forall k\in \left\{1,2,\ldots ,NS\right\}$$

(7) $$\sum_{i=1}^{NT}\sum_{p\in \left\{h,\sigma ,\tau \right\}}x_{impk}\leq NT\times {ws}_{k}\;\forall k\in \left\{1,2,\ldots ,NS\right\},\forall m\in \left\{1,2,\ldots ,NM\right\}$$

(8) $${ws}_{k}\leq \sum_{i=1}^{NT}\sum_{p\in \left\{h,\sigma ,\tau \right\}}x_{impk}\;\forall k\in \left\{1,2,\ldots ,NS\right\},\;\forall m\in \left\{1,2,\ldots ,NM\right\}$$

(9) $$\sum_{i=1}^{NT}{(x}_{imhk}+x_{im\tau k})\leq NT\times h_{k}\;\forall k\in \left\{1,2,\ldots ,NS\right\},\;\forall m\in \left\{1,2,\ldots ,NM\right\}$$

(10) $$h_{k}\leq \sum_{i=1}^{NT}\sum_{p\in \left\{h,\tau \right\}}\left(x_{impk}\right)\;\forall k\in \left\{1,2,\ldots ,NS\right\},\;\forall m\in \left\{1,2,\ldots ,NM\right\}$$

(11) $$w_{ijm}=1-w_{jim}\;\forall i,j\in \left\{1,2,\ldots ,NT\right\},\;\;\forall m\in \left\{1,2,\ldots ,NM\right\},\;i<j$$

(12) $$s_{im}+\sum_{k=1}^{NS}\sum_{p\in \left\{h,\sigma ,\tau \right\}}t_{imp}x_{impk}\leq c\;\forall i\in \left\{1,2,\ldots ,NT\right\},\forall m\in \left\{1,2,\ldots ,NM\right\}$$

(13) $$s_{im}+t_{im\tau }x_{im\tau k}\leq s_{jm}+\psi \left(3-\sum_{p\in \left\{h,\sigma ,\tau \right\}}x_{jmpk}-x_{im\tau k}-w_{ijm}\right)\;\forall \in \left\{1,2,\ldots ,NT\right\},\;\forall m\in \left\{1,2,\ldots ,NM\right\},\;\forall k\in \left\{1,2,\ldots ,NS\right\},\;\;i\neq j$$

(14) $$s_{im}+\sum_{p\in \left\{h,\sigma ,\tau \right\}}t_{imp}x_{impk}\leq s_{jm}+\psi \left(3-\sum_{p\in \left\{h,\sigma ,\tau \right\}}x_{impk}-x_{jm\tau k}-w_{ijm}\right)\;\forall \in \left\{1,2,\ldots ,NT\right\},\forall m\in \left\{1,2,\ldots ,NM\right\},\;\forall k\in \left\{1,2,\ldots ,NS\right\},\;\;i\neq j$$

(15) $$s_{im}+t_{imp}x_{impk}\leq s_{jm}+\psi \left(1-x_{impk}\right)+\psi \left(1-x_{jmpk}\right)+\psi \left(1-w_{ijm}\right)\;\forall i,j\in \left\{1,2,\ldots ,NT\right\},\forall m\in \left\{1,2,\ldots ,NM\right\},\forall k\in \left\{1,2,\ldots ,NS\right\},\;p\in \left\{h,\sigma \right\},\;i\neq j$$

(16) $$\sum_{k=1}^{NS}\sum_{p\in \left\{h,\sigma ,\tau \right\}}{kx}_{impk}\leq \;\sum_{l=1}^{NS}\sum_{p\in \left\{h,\sigma ,\tau \right\}}{lx}_{jmpl}\;\forall i,\;j\in \left\{1,2,\ldots ,NT\right\},\;\forall m\in \left\{1,2,\ldots ,NM\right\},\;e_{ij}=1\;\;$$

(17) $$s_{im}+\sum_{p\in \left\{h,\sigma ,\tau \right\}}t_{imp}x_{impk}\leq s_{jm}+\psi \left(2-\sum_{p\in \left\{h,\sigma ,\tau \right\}}x_{impk}-\sum_{p\in \left\{h,\sigma ,\tau \right\}}x_{jmpk}\right)\;\forall \in \left\{1,2,\ldots ,NT\right\},\forall m\in \left\{1,2,\ldots ,NM\right\},\forall k\in \left\{1,2,\ldots ,NS\right\},\;\;e_{ij}=1$$

(18) $$\sum_{k=1}^{NS}\sum_{p\in \left\{h,\sigma ,\tau \right\}}{kx}_{jmpk}=\sum_{l=1}^{NS}\sum_{p\in \left\{h,\sigma ,\tau \right\}}{lx}_{impl}\;\forall i,\;j\in \left\{1,2,\ldots ,NT\right\},\forall m\in \left\{1,2,\ldots ,NM\right\},\;\rho_{ij}=1$$

(19) $$\sum_{\;p\in \left\{h,\sigma ,\tau \right\}}x_{impk}+\sum_{\;p\in \left\{h,\sigma ,\tau \right\}}x_{jmpk}\;\leq 1\;\forall i,j\in \left\{1,2,\ldots ,NT\right\},\forall k\in \left\{1,2,\ldots ,NS\right\},\forall m\in \left\{1,2,\ldots ,NM\right\},\;\eta_{ij}=1$$

(20) $$\sum_{i=1}^{NT}\left({EE}_{imh}x_{imhk}+{EE}_{im\tau }x_{im\tau k}\right)\leq {EE}_{limit}\;\forall k\in \left\{1,2,\ldots ,NS\right\},\;\forall m\in \left\{1,2,\ldots ,NM\right\}$$

(21) $$s_{\mathrm{im}}\geq 0\;\forall i\in \left\{1,2,\ldots ,NT\right\},\;\forall m\in \left\{1,2,\ldots ,NM\right\}$$

(22) $$x_{impk}\;\epsilon \;\left\{0,1\right\}\;\forall i\in \left\{1,2,\ldots ,NT\right\},\forall m\in \left\{1,2,\ldots ,NM\right\},\forall k\in \left\{1,2,\ldots ,NS\right\},\;\;p\in \left\{h,\;\sigma ,\tau \right\}$$

(23) $$z_{rk}\;\in \;\left\{0,1\right\}\;\forall r\in \left\{1,2,\ldots ,NR\right\},\forall k\in \left\{1,2,\ldots ,NS\right\}$$

(24) $$w_{ijm}\in \left\{0,1\right\}\;\forall i,j\in \left\{1,2,\ldots ,NT\right\},\forall m\in \left\{1,2,\ldots ,NM\right\},\;\;i\neq j$$

(25) $${ws}_{k}\in \left\{0,1\right\}\;\forall k\in \left\{1,2,\ldots ,NS\right\}$$

(26) $$h_{k}\in \left\{0,1\right\}\;\forall k\in \left\{1,2,\ldots ,NS\right\}$$

The objective function (Equation (2)) minimizes the total cost per production cycle of the assembly line by considering fixed-station opening costs, human labor costs, robot-related costs, and the energy consumption associated with robot-assisted operations. Equation (3) ensures that each task is assigned to exactly one station and processed using a single alternative. Equation (4) enforces that tasks requiring a robot or HRC can only be executed if a robot is available at the corresponding station. Equation (5) restricts each robot assignment to one station at most, while constraint (6) guarantees that no station contains more than one robot. Equations (7) and (8) establish the activation status of stations by ensuring that a station becomes active only when at least one task is assigned. Equation (9) states that if a station includes any task executable by a human, either individually or collaboratively, the human presence variable $h_{k}$ must take the value of 1. Equation (10) ensures the reverse condition by requiring that if $h_{k}=1$, then at least one human-executable task must be assigned to that station. Equation (11) enforces all precedence relationships among tasks. Since each station can process only one task at a time by a human, a robot, or through collaboration, these constraints also ensure that a resource cannot handle multiple tasks simultaneously. Equation (12) imposes the cycle time limitation by requiring that the completion time of each task, defined as its start time plus processing duration, does not exceed the cycle time. Equations (13)–(15) regulate conflicts arising from different process alternatives during task sequencing. For collaborative execution, Equations (13) and (14) ensure that no other task in the same station can start before the collaborative task is completed, and collaborative tasks cannot begin before preceding tasks are finished. For non-collaborative execution, constraint (15) ensures that each resource, either human or robot, processes only one task at a time. Equations (16) and (17) further enforce precedence feasibility. Equation (16) ensures that predecessor tasks are assigned either to the same station or to an earlier station. Equation (17) governs task ordering within the same station, ensuring that a successor cannot start before its predecessor is completed. Equations (18) and (19) define zoning requirements among tasks. Equation (18) enforces positive zoning by requiring that compatible and interdependent tasks be assigned to the same station. Conversely, Equation (19) imposes negative zoning by preventing incompatible task pairs from being assigned to the same station. Equation (20) incorporates ergonomic considerations by ensuring that the total workload assigned to each station remains within an acceptable ergonomic limit. Equations (21)–(26) define the domains of the decision variables.

## 4. The Proposed QLHH-GA

This study proposes a Q-learning-based hyper-heuristic genetic algorithm (QLHH-GA) for the straight Ergo-MMALBP-HRC problem. The framework combines evolutionary search with experience-based adaptation to support dynamic decision-making throughout the optimization process. The genetic algorithm evolves a population of candidate solutions, while the Q-learning-based hyper-heuristic adapts the selection of problem-specific low-level heuristics according to the observed search state and the outcomes of previous actions. This integration provides a nature-inspired computational framework in which evolutionary variation and selection are complemented by feedback-driven adaptation.

Genetic algorithms are population-based nature-inspired optimization methods that iteratively improve candidate solutions using selection, crossover, and mutation operators [44]. These mechanisms reflect the evolutionary principles of variation, inheritance, and selective retention, through which populations progressively adapt over successive generations. Their flexibility has led to their application in assembly line balancing problems involving multiple constraints and heterogeneous resources. Nevertheless, conventional implementations generally rely on fixed operator selection strategies, which may limit their adaptability under different search conditions [31].

To address this limitation, the proposed framework incorporates a hyper-heuristic layer for the adaptive selection of problem-specific low-level heuristics during the search process. Hyper-heuristics operate at a higher level of abstraction by selecting appropriate search operators rather than directly modifying candidate solutions. In the proposed framework, the high-level strategy selects low-level heuristics according to the current search state, while the selected heuristic performs the corresponding modification of the candidate solution [45,46]. This hierarchical organization enables the search process to modify its operator-selection behavior in response to the evolving characteristics of the solution population.

A predefined set of problem-specific low-level heuristics is employed to generate candidate solutions. The contribution of each heuristic to the search process is evaluated through reinforcement feedback, allowing effective heuristics to receive greater preference in subsequent decisions. The selection policy therefore develops progressively on the basis of accumulated search experience rather than following a fixed sequence of heuristic applications.

The learning mechanism is implemented through Q-learning, a model-free reinforcement learning method. The search condition is represented as a state, the selected heuristic corresponds to an action, and a reward is assigned based on the resulting changes in solution quality and feasibility. The Q-values are updated iteratively, allowing the method to adjust its selection strategy over time. This state–action–reward cycle establishes a continuous feedback mechanism through which the algorithm learns from previous search outcomes and adapts its subsequent behavior.

The biomimetic foundation of QLHH-GA emerges from the interaction of evolutionary and adaptive-learning principles. At the population level, candidate solutions evolve through variation, recombination, and selective retention. At the decision level, reinforcement feedback shapes heuristic-selection behavior based on accumulated experience. The interaction between these mechanisms produces an adaptive computational system capable of reorganizing its search behavior as optimization conditions change. These biologically inspired principles are transferred to the decision-making structure of the algorithm to address the operational and ergonomic complexity of human–robot collaborative assembly line balancing.

In addition, feasibility is maintained through problem-specific repair and control procedures that ensure compliance with precedence relations, cycle time limits, ergonomic constraints, and resource compatibility. These procedures preserve the problem-specific feasibility of candidate solutions while the evolutionary and learning mechanisms guide the search across the feasible solution space.

The proposed QLHH-GA comprises four main components: solution encoding and decoding procedures, a set of problem-specific low-level heuristics, a Q-learning-based heuristic-selection mechanism, and the integration of these components within the evolutionary search process. The following sections describe each component in detail.

### 4.1. Encoding and Decoding Schemes

In the proposed QLHH-GA framework, each candidate solution is represented using a structured vector-based encoding that captures task assignments, processing mode selections, and robot deployment decisions for the Ergo-MMALBP-HRC problem. The encoding scheme is designed to be compatible with genetic operators while supporting the adaptive selection of low-level heuristics within the Q-learning-based hyper-heuristic mechanism.

Each solution is defined by a fixed-length decision vector composed of three components: x=[S,P,R], where each sub-vector represents a distinct decision dimension of the problem.

The task-to-station assignment vector $S=[S_{1},S_{2},\ldots ,S_{NT}]$ specifies the station to which each task is assigned. For each task i, the value $S_{i}\in \{1,\ldots ,NS\}$ denotes the index of the station where the task is performed.

The processing mode vector $P=[P_{1},P_{2},\ldots ,P_{NT}]$ defines the execution mode of each task. Each element $P_{i}\in \left\{1,2,3\right\}$ corresponds to human-only, robot-only, or human–robot collaborative execution.

The robot allocation vector $R=[R_{1},R_{2},\ldots ,R_{k}]$ represents robot assignment decisions at the station level. Each element $R_{k}\in \{0,\ldots ,NR\}$ indicates whether a robot is assigned to station k, where zero denotes that no robot is deployed.

**Figure 1:**
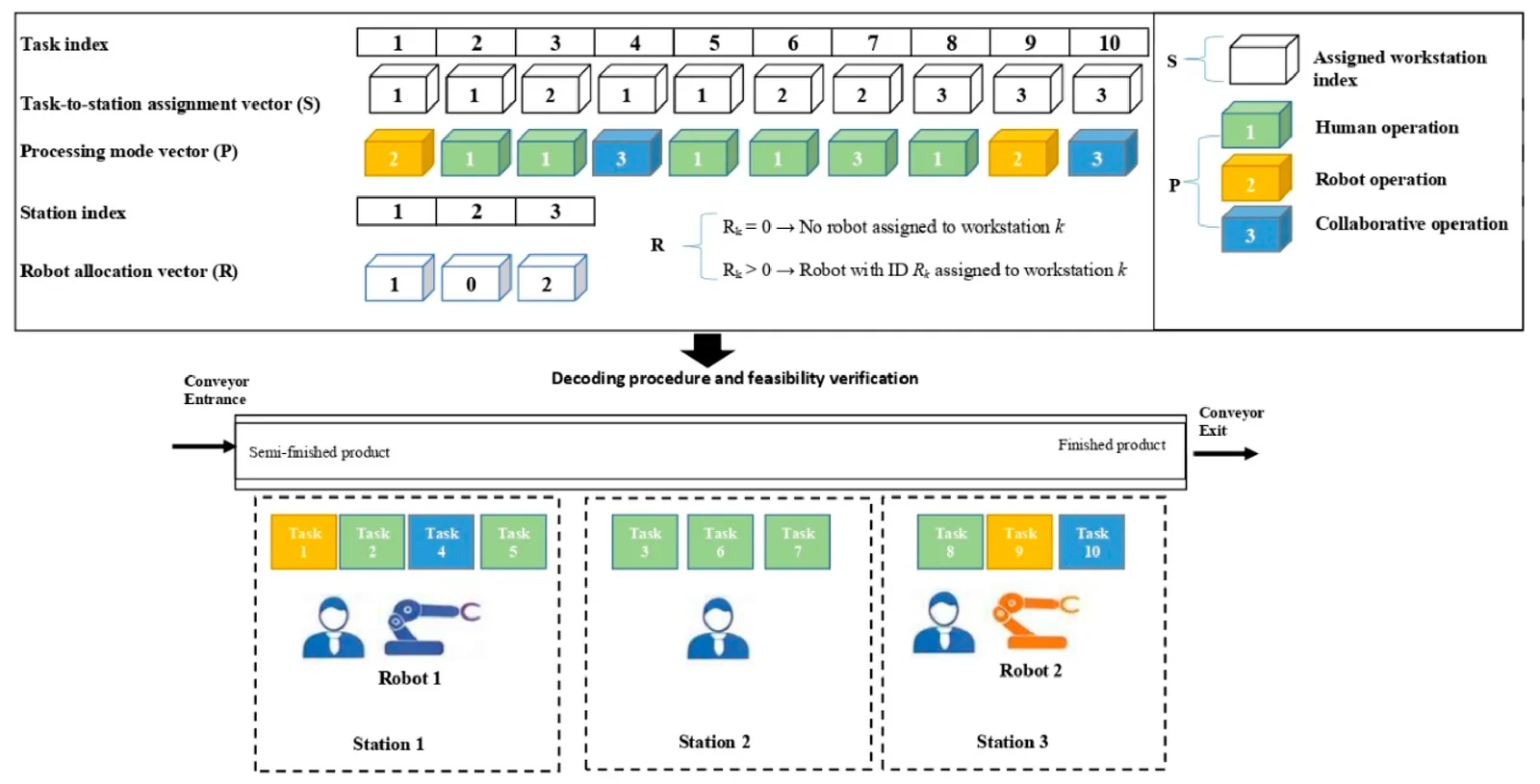
Encoding and decoding scheme of the proposed QLHH-GA.

This representation provides direct and compact mapping between the decision variables and the physical system configuration, while remaining suitable for evolutionary search operations. A schematic illustration of the proposed encoding structure and its relationship with the assembly system is presented in Figure 1.

The decoding procedure transforms the encoded vector into an executable assembly-line configuration. Given a solution x, the decoding process extracts station assignments, processing modes, and robot allocations, and ensures that all decision variables remain within their predefined bounds, as implemented in the solution structure.

Following reconstruction, feasibility is evaluated with respect to precedence constraints, cycle time limits, ergonomic restrictions, and resource compatibility. Infeasible solutions are handled using a repair-based mechanism that improves feasibility without relying solely on penalty functions.

Because human and robot resources may operate independently or collaboratively, the evaluation process includes a station-level scheduling procedure. The tasks assigned to each station are scheduled by considering intra-station precedence relations and potential resource conflicts. Stations operate in parallel, and the completion time at each station must satisfy the cycle time constraint. Therefore, scheduling is treated as an integral part of the decoding and evaluation process, ensuring that each solution corresponds to a feasible and implementable system configuration.

### 4.2. Initial Solution Generation

Initial solutions are generated using a constructive heuristic designed for the Straight Ergo-MMALBP-HRC problem. The procedure considers precedence relations, cycle time constraints, zoning requirements, and human–robot resource compatibility to ensure feasibility.

Tasks are assigned sequentially to stations based on ranked positional weights (${rpw}_{i})$. At each station, precedence-feasible tasks are evaluated, and when a robot is available, robot-compatible tasks are prioritized. For each candidate, the best feasible processing mode is selected based on processing time and resource feasibility.

Stations are filled progressively until no further feasible assignment is possible. After all tasks are assigned, a post-processing step adjusts robot allocations, followed by repair and station-filling procedures to improve feasibility and solution quality. The overall procedure is presented in Algorithm 1.
**Algorithm 1:** Initial solution generation1: Set parameters 2: Initialize all tasks i∈I as unassigned 3: Compute ${\mathrm{rpw}}_{i}$ using precedence matrix IP and minimum processing times 4: Initialize robot allocation vector R 5: Set station index k=1
6: While k≤NS do 7:  Construct candidate list CL (precedence-feasible tasks) 8:  Sort CL in descending order of ${\mathrm{rpw}}_{i}$
9:  If $R_{k}>0$ then 10:   Reorder CL to prioritize robot-compatible tasks 11:  End if 12:  While CL≠∅ do 13:   Initialize a feasibility flag as false 14:   For each task i∈CL do 15:    Determine best feasible processing mode $p_{i}$: 16:    If assigning task i to station k satisfies cycle time and zoning constraints then 17:     Set $S_{i}=k$ and assign processing mode $p_{i}$: 18:     Update station loads 19:     Mark task i as assigned 20:     Set the feasibility flag to true 21:     Break 22:    End if 23:   End for 24:   If the feasibility flag remains false then 25:    Break 26:   End if 27:  End while 28:  Set k=k+1 29: End while 30: If any task remains unassigned, return infeasible 31: Adjust robot allocations through post-processing 32: Apply repair procedure 33: Apply empty-station filling procedure 34: Return solution

### 4.3. Low-Level Heuristics (LLHs)

In the proposed QLHH-GA framework, the search process is guided by a set of low-level heuristics (LLHs) that transform an incumbent solution into a new candidate. The performance of the hyper-heuristic is therefore strongly influenced by the design of these LLHs, their diversity, and the selection mechanism [47].

In this study, the LLHs are designed as efficient neighborhood operators targeting the main decision components of the Ergo-MMALBP-HRC problem, including task-to-station assignment, process alternative selection, and robot allocation. Given the varying exploration and exploitation capabilities of different operators, a Q-learning mechanism adaptively selects LLHs based on their historical performance.

Each LLH modifies one or more components of the solution vector, followed by a repair-and-check procedure to ensure feasibility. Cycle time violations are corrected through a dedicated repair mechanism, while precedence and zoning constraints are strictly enforced. Furthermore, robot–process compatibility constraints are explicitly checked, ensuring that robot-dependent processing modes are only assigned to stations with an available robot. In addition, a station-level scheduling procedure is applied to verify intra-station precedence and resource conflicts. Solutions that remain infeasible after the repair stage are discarded, and the incumbent solution is preserved. The proposed low-level heuristics are defined as follows:

Minimum processing time-based reassignment (LLH1): A task with the shortest processing time is selected and reassigned to a feasible station to improve station utilization and support a finer redistribution of workload.

Maximum processing time-based reassignment (LLH2): A task with the longest processing time is selected and relocated to reduce workload concentration and alleviate potential bottlenecks.

Maximum successor-based reassignment (LLH3): A task with a large number of successor tasks is selected and reassigned to improve the precedence structure and strengthen task flow consistency.

Minimum successor-based reassignment (LLH4): A task with a small number of successor tasks is selected and reassigned to provide greater flexibility while limiting disruption to the overall precedence structure.

Maximum positional weight-based reassignment (LLH5): Tasks are ranked according to their positional weights, defined by their processing times and successor-related contributions, and the highest-ranked task is reassigned to a feasible station.

Random task reassignment (LLH6): A randomly selected task is reassigned to a feasible station to enhance diversification during the search.

Bottleneck station load reduction (LLH7): A task assigned to the most heavily loaded station is selected and reassigned to another feasible station to reduce cycle-time pressure and improve workload balance.

Adjacent station relocation (LLH8): A task is moved to an adjacent upstream or downstream station to introduce a controlled local perturbation while preserving the overall station structure.

Processing mode modification (LLH9): The processing mode of a selected task, namely human, robot, or collaborative execution, is changed to another feasible alternative while respecting station-level robot availability constraints.

Robot allocation adjustment (LLH10): Robot assignments across stations are modified to improve resource compatibility and reduce operational cost by swapping or redistributing robot assignments among stations.

Task reassignment with simultaneous mode adaptation (LLH11): A task is reassigned to a different station, and its processing mode is simultaneously updated to maintain feasibility and improve solution quality.

Multi-task stochastic perturbation (LLH12): A subset of tasks is randomly selected and reassigned to different stations, with processing modes updated when necessary, to promote broader exploration of the solution space.

### 4.4. Q-Learning-Based High-Level Strategy

A Q-learning–based high-level strategy is incorporated into the proposed algorithm to improve adaptability and guide the selection of LLHs. Q-learning is a model-free reinforcement learning method that enables the learning of decision policies through interaction with the search process. Owing to its simplicity and stability, it has been applied in various hyper-heuristic optimization studies [5].

The considered problem involves a dynamic search space due to precedence relations, station capacity limits, ergonomic constraints, and mixed-model characteristics. Under such conditions, static or random LLH selection may lead to inefficient search behavior. The integration of Q-learning allows the algorithm to adaptively select LLHs based on the current solution state.

The Q-learning mechanism is defined by five main components: state, action, action selection strategy, reward function, and update rule. These components are structured in accordance with the search process and the characteristics of the assembly line balancing problem. The overall procedure follows the Bellman optimality principle and is summarized in Algorithm 2.
**Algorithm 2:** The Q-learning algorithmInput: Current solution $x_{t}$, best solution found so far $x^{*}$, Q-table *Q*, action set *A*, learning rate *λ*, discount factor γ, exploration parameters $\varepsilon_{0}$, $\varepsilon_{\min}$, δ Output: updated Q-table *Q*1: Initialize $Q\left(s,a\right)=0$ for all states s∈S and actions a∈A. 2: Initialize exploration rate $\varepsilon =\varepsilon_{0}$. 3: Determine the current state $s_{t}$ based on the feasibility, balance, and stagnation status of $x_{t}$. 4: While the termination criterion is not met do 5:  Select an action $a_{t}$ using the ε-greedy defined in Equation (27) 6:  Apply $a_{t}$ to $x_{t}$ to obtain $x_{t+1}$; repair/evaluate and compute $F_{new}$. 7:  Compute the reward $r_{t}$ according to Equations (29) and (30) 8:  Determine the next state $s_{t+1}$ from $x_{t+1}.$ 9:  Update $Q_{t}\left(s_{t},a_{t}\right)$ using Equation (31). 10:  Update the exploration rate $\varepsilon_{t+1}\leftarrow \max\left(\varepsilon_{min},\varepsilon_{t}.\delta \right)$ as defined in Equation (28). 11.      Set t←t+1, $x_{t}\leftarrow x_{t+1}$, ${s_{t}\leftarrow s}_{t+1}$. 12: End while 13: Return *Q*.

#### 4.4.1. Definition of State

The state represents the current search condition of a candidate solution within the population and serves as the basis for adaptive operator selection in the proposed Q-learning framework. Rather than employing a high-dimensional or problem-specific state representation, a compact and structured discrete state space is defined to balance learning effectiveness with computational efficiency.

Let $s_{t}\in S$ denote the state of a solution at iteration t. In contrast to conventional formulations that primarily rely on feasibility and improvement indicators, the state definition in this study incorporates violation characteristics, workload distribution, and search progress dynamics. Accordingly, eight distinct states are defined:

State 1 (Cycle-time infeasible): The solution violates cycle time constraints while satisfying ergonomic limits.

State 2 (Ergonomically infeasible): The solution violates ergonomic constraints without exceeding cycle time limits.

State 3 (Fully infeasible): The solution simultaneously violates both cycle time and ergonomic constraints.

State 4 (Feasible but highly unbalanced): The solution is feasible; however, a high imbalance exists among station workloads, indicating poor load distribution.

State 5 (Feasible and moderately balanced): The solution is feasible with a moderate level of workload imbalance, suggesting potential for further improvement.

State 6 (Feasible and well-balanced): The solution is feasible and exhibits a balanced workload distribution across stations.

State 7 (Improvement state): The solution yields an improvement over either the current solution or the global best solution, indicating a successful exploitation phase.

State 8 (Stagnation state): The solution does not show improvement after a predefined number of consecutive iterations, indicating search stagnation.

This state formulation enables the learning mechanism to differentiate between feasible and infeasible regions and among distinct sources of infeasibility and varying levels of solution quality. Furthermore, by explicitly capturing workload balance and stagnation behavior, the proposed state space supports the adaptive selection of low-level heuristics based on the current search condition. This structure helps the search process respond more effectively to different solution space regions.

#### 4.4.2. Definition of Action

In the proposed framework, an action corresponds to selecting a low-level heuristic (LLH) from the heuristic pool introduced in Section 4.3. Let $A=\left\{a_{1},a_{2},{\ldots ,a}_{12}\right\},$ where each action represents a specific neighborhood operator that modifies the current solution through changes in task assignment, processing mode, or robot allocation.

At each decision step, the Q-learning agent selects an LLH according to its current policy and applies it to the current solution. The resulting solution is then subjected to local search, repair, and feasibility checks and evaluated using the fitness function.

The impact of the selected action is assessed based on the change in the solution quality, which is used to update the corresponding state–action value. Accordingly, the learning mechanism promotes the selection of more effective heuristics under various search conditions.

#### 4.4.3. Selection Strategy of Action

An ε-greedy policy is employed to balance exploration and exploitation during the learning process, following Q-learning-based hyper-heuristic approaches in Zhang et al. [48]. At iteration t, a random number $p\in \left[0,1\right]$ is generated. If $p\leq \varepsilon_{t},$ an action is selected uniformly at random from the action set A to promote exploration. Otherwise, the action with the highest Q-value in the current state is selected to exploit the accumulated search experience. When multiple actions share the same maximum Q-value, one of them is selected randomly. Accordingly, the selected action $a_{t}$ is determined as follows [48]:

(27) $$a_{t}=\;\left\{\begin{array}{c} \mathrm{random}\;a\;\mathrm{in}\;A,\;\;\;\mathrm{if}\;p\leq \;\varepsilon_{t},\\ {arg\;max}_{a\;\epsilon \;A}\;Q\;\left(s_{t},\;a\right),\;\;\mathrm{otherwise}. \end{array}\right.$$

The exploration rate is updated using an exponentially decaying scheme [49]:

(28) $$\varepsilon_{t+1}=\max\left(\varepsilon_{min},\delta \cdot \varepsilon_{t\;}\right)$$

where $\varepsilon_{0}$ is the initial exploration probability, $\varepsilon_{min}$ is the minimum exploration level, and δ∈(0,1) is the decay coefficient.

This mechanism enables broader exploration in the early stages of the search while gradually favoring high-performing actions.

#### 4.4.4. Reward Function

The reward function is a key component of the Q-learning-based high-level strategy, as it directs the learning process by reinforcing effective actions under different search conditions. Its formulation influences the ability of the agent to learn desirable search behavior and affects the overall convergence of the algorithm [5].

The reward structure is designed to support feasibility recovery, encourage improvements in the objective function, and promote the identification of improved solutions. To ensure consistent behavior, infeasible solutions are represented by non-finite fitness values. Let $F_{old}$ and $F_{new}$ denote the fitness values before and after applying action $a_{t}$, and let $F_{best}$ be the best (lowest) fitness obtained so far. The base reward ${\bar{r}}_{t}$ is defined as follows:

(29) $${\bar{r}}_{t}=\left\{\begin{array}{c} 3,\;\;\;\mathrm{if}\;F_{old}\;\mathrm{is}\;\mathrm{infeasible}\;\mathrm{and}\;F_{new}\;\mathrm{is}\;\mathrm{feasible};\\ -1,\;\;\;\mathrm{if}\;F_{old}\;\mathrm{is}\;\mathrm{infeasible}\;\mathrm{and}\;F_{new}\;\mathrm{is}\;\mathrm{infeasible};\\ -2,\;\;\mathrm{if}\;F_{old}\;\mathrm{is}\;\mathrm{feasible}\;\mathrm{and}\;F_{new}\;\mathrm{is}\;\mathrm{infeasible};\\ 1+\min\left(2,{(F}_{old}-F_{new})/\max\left(F_{old},\kappa \right)\right),\;\mathrm{if}\;F_{new}<F_{old};\\ -0.5,\;\;\;\mathrm{otherwise}. \end{array}\right.$$

where κ a small positive constant ($\kappa =10^{-9}$) is introduced to avoid division by zero. The term ($F_{old}-F_{new}$) reflects the magnitude of improvement.

In addition to the base reward, an extra reward is assigned when the applied action produces a new global best solution. Accordingly, the final reward is defined as:

(30) $$r_{t}={\bar{r}}_{t}+I(F_{new}<F_{best})$$

where I(⋅) is the indicator function.

#### 4.4.5. Update Mechanism

The Q-table maintains the experience accumulated through the interaction between the agent and the search process. Each entry $Q_{t}\left(s_{t},a_{t}\right)$ reflects the estimated utility of selecting action $a_{t}\in A$ when the system is in state $s_{t}\in S$. After applying an action, observing the immediate reward $r_{t},$ and identifying the subsequent state $s_{t+1}$, the Q-value is updated using a temporal-difference learning rule.

In this study, the update follows an incremental formulation, where λ∈[0,1] is the learning rate and γ∈[0,1] is the discount factor. The Q-value update is given by [48]:

(31) $$Q_{t+1}\left(s_{t},a_{t}\right)=(1-\lambda )Q_{t}\left(s_{t},a_{t}\right)+\lambda \left[r_{t}+\gamma {max}_{a\;\in \;A}Q\left(s_{t+1},a\;\right)\right]$$

This update mechanism adjusts the current estimate toward a target value that combines the immediate reward and the expected future return, thereby enabling the progressive refinement of action preferences.

### 4.5. The Framework of Q-Learning-Based Hyper-Heuristic Genetic Algorithm

The proposed solution approach is based on the QLHH-GA, where a genetic algorithm performs the population-based search and low-level heuristics are selected adaptively through a Q-learning mechanism. This structure enables the integration of population-based exploration with state-dependent solution refinement.

#### 4.5.1. Population Initialization

The initial population is generated using the constructive procedure described in Algorithm 1. Each individual represents a complete solution encoding task-to-station assignments, processing mode selections, and robot allocation decisions.

All generated solutions are evaluated regarding precedence relations, cycle time, ergonomic constraints, and resource compatibility. Infeasible solutions are repaired whenever possible. The best feasible solution identified during initialization is stored as the initial global best solution $x^{*}$.

#### 4.5.2. Selection Mechanism

Parent solutions are selected using tournament selection, which represents the competitive selection mechanism of the bio-inspired evolutionary framework. A subset of individuals is randomly sampled from the population, and the solution with the best fitness value is selected as a parent. This mechanism maintains a balance between selection pressure and population diversity.

#### 4.5.3. Crossover Operator

A crossover operator is applied to generate offspring solutions by recombining the decision variables of parent individuals. The operator preserves structural consistency of the solution representation while enabling information exchange between individuals. This process contributes to the exploration of new regions in the search space.

#### 4.5.4. Mutation Operator

Mutation is applied to introduce controlled perturbations into offspring solutions. Selected components of the solution representation are modified to increase diversity and reduce the risk of premature convergence. The resulting solutions are subsequently evaluated with respect to feasibility conditions.

#### 4.5.5. Offspring Generation and Improvement Procedure

Following crossover and mutation, each offspring solution is further improved using a Q-learning-guided procedure. For a given offspring, the current state $s_{t}$ is determined according to the state definition provided in Section 4.4.1.

An action $a_{t}$, corresponding to a low-level heuristic, is selected using the ε-greedy strategy defined in Section 4.4.3. The selected heuristic is applied to the offspring solution, followed by a local search procedure. The resulting candidate solution is then evaluated using the fitness function.

The observed change in solution quality is used to compute the reward value, and the Q-table is updated according to the update mechanism described in Section 4.4.5. This process enables the adaptive reinforcement of effective heuristics under different search conditions.

#### 4.5.6. Replacement Strategy and Elitism

A greedy acceptance mechanism is applied at the solution level. If the candidate solution improves the fitness value of the current offspring, it is accepted; otherwise, the original solution is retained.

After all offspring solutions are processed, the population is updated by selecting the best individuals based on fitness values. Elitism is applied to preserve high-quality solutions across iterations, reflecting the survival and inheritance of advantageous solution characteristics. The global best solution $x^{*}$ is updated whenever a better feasible solution is obtained.

#### 4.5.7. QLHH-GA Framework

The complete procedure of the proposed QLHH-GA is presented in Algorithm 3.
**Algorithm 3:** QLHH-GA1: Set algorithm parameters (population size, crossover and mutation probabilities, elitism rate, learning parameters, and termination criterion) 2: Initialize Q(s, a) = 0  and set $\varepsilon =\varepsilon_{0}$ 3: Generate NP initial solutions using Algorithm 1 4: Evaluate all solutions 5: Store the best solution as $x^{*}$ 6: while $\mathrm{elapsed}\;\mathrm{time}<T_{\max}$ do 7:  Select parent solutions using tournament selection 8:  Apply crossover and mutation to generate offspring 9:  for each offspring solution do 10:   Determine current state $s_{t}$
11:   Select action $a_{t}$ using ε-greedy policy defined in Equation (27) 12:   Apply the selected low-level heuristic followed by a local search procedure 13:   Evaluate the candidate solution 14:   If the candidate improves the current solution, accept it; otherwise retain the original solution 15:   Compute the reward and update the Q-table 16:   Update $x^{*}$ if a better solution is obtained 17:  end for 18:  Update $\varepsilon \leftarrow \max(\varepsilon_{\min},\;\varepsilon \cdot \delta )$
19: end while 20: return $x^{*}$


## 5. Computational Experiments

This section evaluates the proposed MILP formulation and QLHH-GA using benchmark instances of varying sizes and a literature-based industrial case study. Section 5.1 describes the experimental settings, and Section 5.2 presents the parameter-tuning procedure. Section 5.3 reports the computational results, including comparisons with the MILP model and benchmark metaheuristics, an ablation analysis, statistical tests, and the case study.

### 5.1. Experimental Settings

The performance of the proposed straight-line Ergo-MMALBP-HRC model is evaluated using benchmark instances widely adopted in the assembly line balancing literature. The datasets are obtained from the ALBP database (assembly-line-balancing.de) and extended to incorporate HRC and ergonomic parameters. The corresponding data are provided as Supplementary Materials.

The MILP model is solved using IBM ILOG CPLEX Optimization Studio 22.1.1.0 with a time limit of 1800 s per run. The proposed QLHH-GA and benchmark metaheuristics are implemented in MATLAB R2024a. All computational experiments were conducted on a computer equipped with an Intel Core i5-8250U processor at 1.60 GHz, 8 GB of RAM, and a 64-bit Windows 10 operating system. The algorithms were executed sequentially without parallelization.

To evaluate performance under different problem scales, the instances are categorized based on the number of tasks (NT) as small (NT≤20), medium (20<NT≤69), and large (NT≥70). Additional parameters required for the HRC context are generated and integrated into the benchmark structures.

Three processing modes are considered for each task: human-only, robot-only, and collaborative. Processing times follow the commonly adopted assumptions in the literature [23,50], where collaborative execution yields shorter times than human-only processing, while robot-only execution is generally slower due to operational constraints [12,51].

The processing time of task i of model m performed by a human is denoted as $t_{\mathrm{imh}}$. The robot processing time is defined as $t_{im\sigma }=t_{\mathrm{imh}}\cdot U(1.3,1.4)$, and the collaborative processing time is given as $t_{im\tau }=t_{imh}\cdot U(0.6,0.7)$.

The ergonomic risk for human execution, denoted ${\mathrm{EE}}_{\mathrm{imh}},$ is generated using a uniform distribution U(0.1,4.0). For collaborative execution, the ergonomic risk is computed as ${\mathrm{EE}}_{\mathrm{im}\tau }={EE}_{\mathrm{im}h}\cdot U\left(0.3,0.8\right)$, following Stecke and Mokhtarzadeh [23].

The maximum allowable ergonomic risk per station (${EE}_{limit}$) is computed based on the worst-case task-level ergonomic loads and scaled using a variability factor ξ∼U(1.0,1.3), as given in Equation (32). This formulation ensures balanced ergonomic exposure across stations.

(32) $${EE}_{limit}=\left(\frac{1}{NS}\sum_{i=1}^{NT}{{max}_{m\in \left\{1,\;2,\ldots ,\;\;NM\right\}\;}\mathrm{EE}}_{\mathrm{imh}}\right)\cdot \xi$$

Cost parameters are incorporated to evaluate operational performance. The station cost is set to 1.4 €/time unit, and the human labor cost is 0.7 €/time unit when applicable. Robot-related cost parameters are generated from uniform distributions. The cost of robot r, denoted by ${cr}_{r}$, follows $U\left(0.08,0.15\right)$, while the unit-time energy consumption cost ${ce}_{r}$ is drawn from U(0.15,0.45). These settings reflect variability in robot-related costs and are adapted from Nourmohammadi et al. [10] and Yilmaz et al. [20].

The performance of the proposed QLHH-GA is compared with the exact MILP model and three benchmark metaheuristics: GA, PSO, and ABC. All algorithms employ the same solution representation and are initialized using Algorithm 1 to ensure fair comparison. The implementation of benchmark algorithms follows standard frameworks reported in the literature, including Li et al. [52].

All algorithms are executed under identical computational settings. A time-based stopping criterion is applied, where the maximum runtime is defined as $T_{max}=NT\cdot NT\cdot \tau$ milliseconds. To evaluate performance under different computational budgets, τ is set to 60, 80, and 100. Each algorithm is independently executed ten times, and the best solution is reported.

### 5.2. Parameter Tuning

The performance of metaheuristic algorithms is sensitive to the selection of control parameters; therefore, a systematic tuning procedure was conducted before the computational experiments. The Taguchi method was employed to calibrate the main parameters of the algorithms, while secondary parameters were fixed at commonly used values reported in the literature [2,53].

The Taguchi approach uses orthogonal experimental designs to efficiently explore the parameter space with a limited number of experiments [54]. In this study, tuning was performed on a representative large-size instance, as large-scale problems better reflect the search behavior of metaheuristics.

A three-level Taguchi design was adopted for each algorithm based on the control factors given in Table 3. Accordingly, an L9 orthogonal array was used, resulting in nine parameter configurations per algorithm. Each configuration was evaluated over ten independent runs, and the mean objective value was used as the response for the Taguchi analysis conducted using Minitab Statistical Software (version 22.5.1).

**Figure 2:**
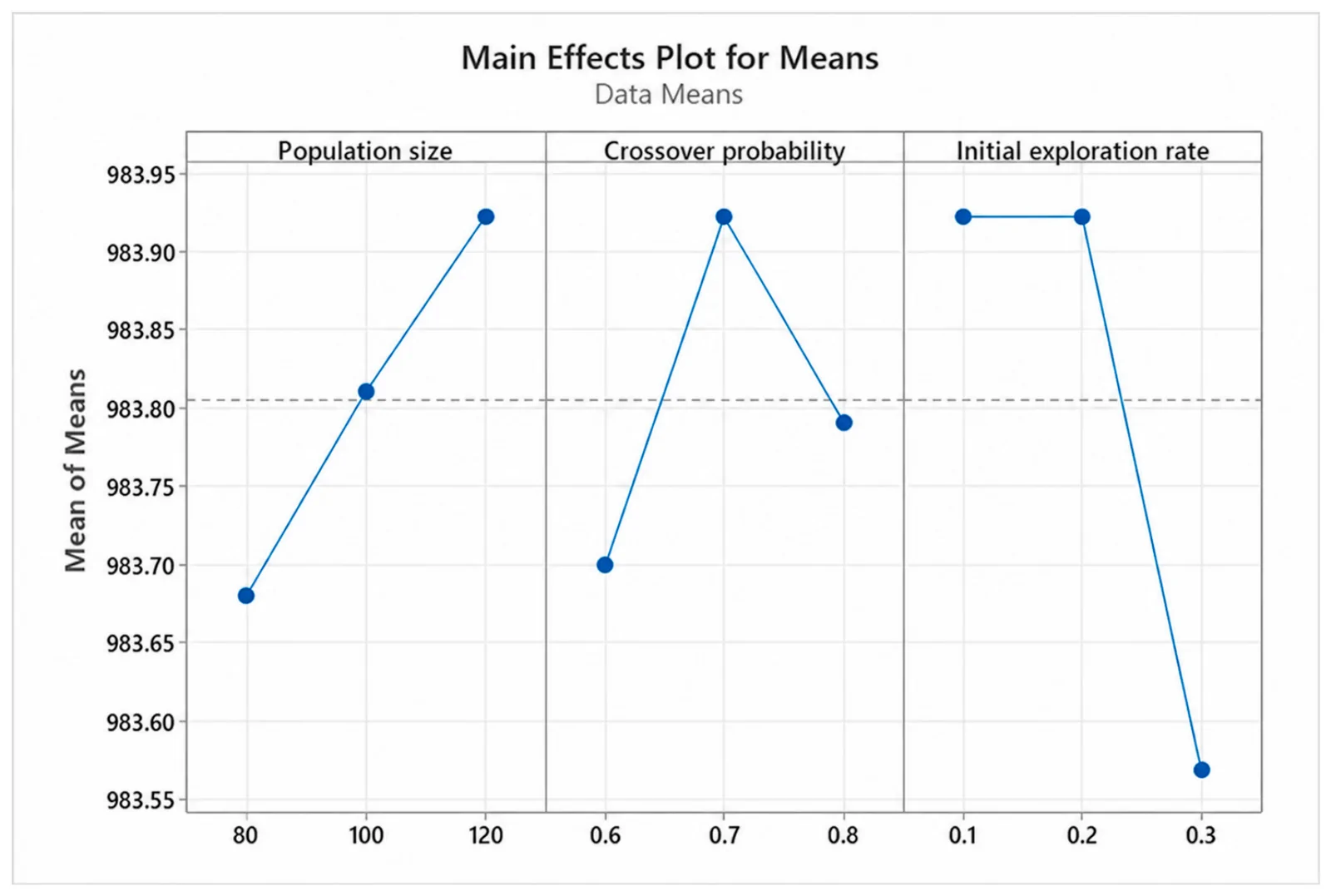
Main effects of QLHH-GA parameters on the mean objective value. The dashed horizontal line indicates the overall mean objective value.

**Figure 3:**
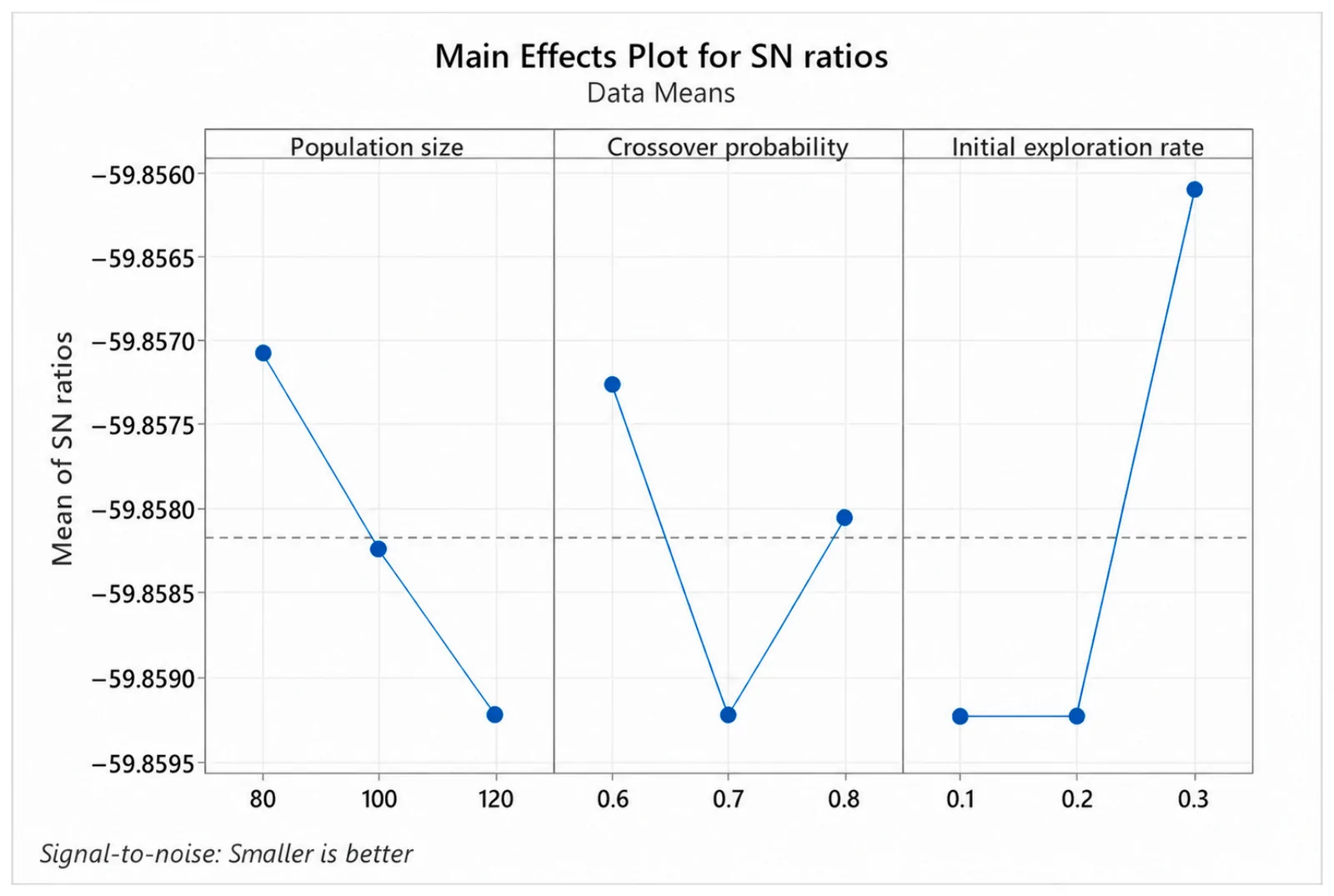
Main effects of QLHH-GA parameters on the signal-to-noise ratio. The dashed horizontal line indicates the overall mean signal-to-noise ratio.

The signal-to-noise (S/N) ratio was used as the primary criterion for selecting robust parameter settings, while mean performance served as a secondary measure. The main effects plots for the mean response and the S/N ratios obtained for the parameter tuning of the proposed QLHH-GA are presented in Figure 2 and Figure 3, respectively. Based on the S/N ratio analysis, the parameter combination of NP=80, $P_{c}=0.6$, and $\varepsilon_{0}=0.3$ was selected as the final configuration because it provided the most robust performance. The final parameter settings for the QLHH-GA and benchmark algorithms are reported in Appendix A Table A1.

### 5.3. Computational Results

#### 5.3.1. Comparison of the MILP and QLHH-GA

The performance of the proposed QLHH-GA was compared with that of the MILP formulation solved using IBM ILOG CPLEX under the experimental framework described in Section 5.1. The detailed results are reported in Table 4, where the total cost, maximum EE, computational time, and optimality gap for the MILP model are presented alongside the best solutions obtained by QLHH-GA. The relative percentage deviation (RPD) metric is used to quantify the performance gap between the two approaches.

The RPD is calculated using Equation (33):

(33) $$RPD=\;100\times (C{ost}_{alg}-{Cost}_{best})/{Cost}_{best}$$

The RPD is computed by taking the MILP objective value as the reference. Accordingly, positive RPD values indicate inferior solutions obtained by QLHH-GA, whereas negative values imply that the proposed method outperforms the best incumbent solutions identified by the MILP solver within the imposed time limit.

For small-sized instances (P1–P3), the MILP model achieves optimal or near-optimal solutions with negligible optimality gaps, reflecting the tractability of these instances for exact optimization. Under these conditions, QLHH-GA exhibits competitive performance. It reproduces the MILP solutions in all P3 configurations and in the zero-robot configuration of P1, while the remaining P1 deviations remain below 1%. In the P2 instances, moderate deviations are observed, with RPD values reaching up to approximately 9%. Nevertheless, these differences remain within an acceptable range for metaheuristic approaches, considering the absence of optimality guarantees.

From an ergonomic standpoint, both approaches consistently satisfy the predefined energy expenditure constraints. Furthermore, the observed variations in EE values between the MILP and QLHH-GA solutions suggest that multiple feasible workload distributions exist, allowing the algorithm to explore alternative configurations without violating ergonomic limits.

For medium-sized instances (P4, P5, and P6), the increasing combinatorial complexity is reflected in the non-zero optimality gaps reported by the MILP model. Under these conditions, the relative performance of QLHH-GA becomes more evident. While certain instances exhibit marginally higher cost values, the proposed method can identify superior solutions in multiple configurations. In particular, negative RPD values observed in instances such as P4 (−0.41) and P6 (−7.19) indicate that QLHH-GA can surpass the best incumbent solutions obtained by CPLEX within the specified time limit. The improvement is especially notable in the larger configurations of the P6 instance, where the search space becomes significantly more complex.

The analysis of maximum EE values further supports the robustness of the proposed approach. In Instances 22 and 24, the lower cost obtained by QLHH-GA is accompanied by a lower maximum EE value than that of the MILP solution. In other configurations, EE values remain comparable, suggesting that the algorithm maintains a stable workload distribution while exploring cost-efficient solutions.

An additional insight emerging from Table 4 concerns the systematic impact of robot allocation on both cost and ergonomic performance. The configurations with greater robot availability are evaluated at lower cycle-time levels and generally produce lower total costs. Given that the objective function minimizes the total cost per production cycle and that the cost components are scaled by the cycle time coefficient, reductions in cycle time result in a proportional decrease in the total cost. This behavior is observed across multiple instances, particularly in P1, P2, and P6, where higher robot availability corresponds to lower cost values.

The maximum EE values remain below the prescribed limits in all configurations. Although additional robots are associated with lower maximum EE values in several instances, this relationship is not strictly monotonic because ergonomic performance also depends on the resulting task and processing-mode assignments.

Overall, the computational results indicate that QLHH-GA achieves solution quality comparable to MILP for small-sized instances and demonstrates superior performance on medium-sized problems where exact methods are constrained by computational limitations. Furthermore, the observed interaction between robot allocation, cycle time reduction, cost minimization, and energy expenditure confirms that the proposed model effectively captures the underlying trade-offs between economic and ergonomic objectives. These findings highlight the effectiveness of the proposed learning-based hyper-heuristic framework in addressing realistic HRC assembly line balancing problems.

#### 5.3.2. Comparison of QLHH-GA with Benchmark Metaheuristics

The performance of the proposed QLHH-GA was evaluated through a comparative analysis with three widely used population-based metaheuristics, namely, GA, PSO, and ABC. These algorithms are frequently adopted as benchmark methods in assembly line balancing studies and provide a suitable basis for assessing the effectiveness of advanced search frameworks. To ensure consistency, all algorithms were implemented using the same solution encoding, decoding, and feasibility evaluation procedures.

The instance-level results obtained for *τ* = 100 are reported in Table 5. For each configuration, the best objective value, the corresponding maximum energy expenditure, and the RPD values are presented. The RPD values are calculated by taking the best objective value obtained among all algorithms for each instance as the reference. In addition, Table 6 reports the average RPD values across different τ levels, allowing the evaluation of algorithmic performance under varying computational time limits.

For P1 and P2, the algorithms generally produce similar objective values, although moderate differences occur in several robot configurations. More pronounced differences are observed for P3, where the RPD reaches 8.09% for GA, 6.85% for PSO, and 4.49% for ABC. In these cases, QLHH-GA consistently achieves the best solutions, with RPD values equal to zero across all configurations. This outcome indicates that the solution space of smaller instances can be effectively explored within the available computational time by all considered methods. From an ergonomic perspective, all algorithms generate solutions that satisfy the predefined EE constraints, and the variations in maximum EE values remain limited. This suggests that multiple feasible configurations exist that simultaneously achieve cost efficiency and ergonomic compliance.

As problem size increases, more pronounced differences in performance emerge. For medium-sized instances (P4–P6), QLHH-GA consistently achieves the best or near-best solutions across almost all configurations, maintaining zero or minimal RPD values. In contrast, GA, PSO, and ABC exhibit higher deviations, with RPD values increasing in several instances. These differences become more evident in configurations with a higher number of robots, where the interaction between task assignment, processing mode selection, and resource allocation increases the complexity of the search space.

The results indicate that QLHH-GA shows stable performance across different problem settings. While the competing algorithms show variation in solution quality, QLHH-GA maintains a consistent level of performance. This behavior can be attributed to the adaptive operator selection mechanism, which enables the algorithm to dynamically adjust its search strategy based on the evolving solution state. Consequently, the search process is guided more effectively toward high-quality regions of the solution space.

With respect to ergonomic performance, all algorithms produce solutions that remain within the allowable EE limits, confirming feasibility. However, QLHH-GA tends to maintain a more consistent distribution of workload, as reflected in the maximum EE values. In some instances, competing algorithms achieve slightly lower EE values but at the expense of higher operational costs. In contrast, QLHH-GA maintains ergonomic feasibility while achieving the lowest operational cost. This indicates that the proposed approach effectively manages the interaction between workload distribution and cost minimization.

The aggregated results presented in Table 6 further support these observations. Across different τ values, QLHH-GA consistently yields the lowest average RPD values, indicating superior robustness under varying computational budgets. Although increasing τ improves the performance of all algorithms, the relative advantage of QLHH-GA remains evident, particularly for medium-sized instances. This suggests that the proposed method is less sensitive to time limitations and is capable of maintaining solution quality even under constrained computational conditions.

The overall findings indicate that QLHH-GA provides a reliable and effective solution approach for the considered problem. The integration of a learning-based selection mechanism within the hyper-heuristic framework enhances adaptability and improves solution quality, particularly in instances characterized by complex interactions among cost, cycle time, and energy expenditure. Furthermore, the consistent satisfaction of ergonomic constraints demonstrates that performance improvements are achieved without compromising worker-related considerations.

#### 5.3.3. Ablation Analysis of Q-Learning-Based LLH Selection

An ablation analysis was conducted to assess the contribution of Q-learning-based low-level heuristic (LLH) selection. QLHH-GA was compared with a variant using random LLH selection at *τ* = 100, while all other algorithmic components and computational settings were kept unchanged. The analysis considered the largest robot configuration of each large-sized problem (P7–P9). Mean-cost improvement was calculated relative to random LLH selection, while Maximum EE (Best) represents the lowest maximum station-level EE obtained across the independent executions.

Table 7 shows that Q-learning-based LLH selection achieved lower mean and best costs for all three problems. The mean-cost improvements were 0.220%, 0.513%, and 3.715% for P7, P8, and P9, respectively. These results indicate that Q-learning-based LLH selection improves cost performance and consistency compared with random selection, with the largest improvement observed for P9.

#### 5.3.4. Statistical Analysis

A non-parametric statistical analysis was conducted to assess performance differences among the algorithms. The analysis was based on the RPD values reported in Table 5 for *τ* = 100. Each algorithm was independently executed ten times for every instance–robot configuration, and the best objective value obtained across these runs was used to calculate the corresponding RPD. The normality assumption was not satisfied for the performance data, as indicated by the normality tests. Therefore, the Friedman test was employed to determine whether statistically significant differences exist among the algorithms.

The results of the Friedman test show a statistically significant difference among the methods ($\chi^{2}=62.70$, df=3, p<0.001). The mean ranks indicate that QLHH-GA achieves the best overall performance (1.51), followed by ABC (2.12), GA (3.00), and PSO (3.25). This ranking indicates that the proposed approach achieves the best overall performance across the instances considered.

Pairwise differences were further examined using the Nemenyi post hoc test, and the results are reported in Table 8. The analysis reveals statistically significant differences between QLHH-GA and both GA (p<0.001) and PSO (p<0.001). In contrast, the difference between QLHH-GA and ABC (p=0.082) is not statistically significant at the 0.05 level, although QLHH-GA exhibits a lower mean rank. This finding suggests that, while the proposed method demonstrates a consistent advantage over ABC, the difference cannot be confirmed statistically under the selected significance level.

Additional comparisons show that ABC performs better than PSO (p=0.005). However, the differences between GA and PSO (p=0.844) and between GA and ABC (p=0.058) are not statistically significant. These results imply that the relative performance differences among the benchmark algorithms are not consistently strong across all pairwise comparisons.

The overall statistical evidence supports the effectiveness of the proposed QLHH-GA. The method achieves the best mean rank and demonstrates statistically significant improvements over GA and PSO. Although the difference with ABC is not statistically significant, QLHH-GA maintains a more favorable ranking, indicating a consistent performance advantage across the benchmark instances.

#### 5.3.5. Case Study

This section presents a literature-based industrial case study to evaluate the applicability of the proposed model and solution approach. The case is based on an industrial assembly system from the automotive sector, reported by Nourmohammadi, Fathi, and Ng [10] and extended using the ergonomic modeling framework of Cai et al. [26]. The system involves the assembly of a mass balancing system (MBS), which represents a typical product requiring coordinated manual and automated operations under HRC.

The assembly line consists of technology-enabled workstations where tasks can be executed in three modes: human-only, robot-only, or collaborative human–robot execution. Such configurations reflect contemporary Industry 4.0 and Industry 5.0 manufacturing environments, where humans and collaborative robots operate within shared workspaces to jointly perform assembly operations. Depending on the process requirements and resource availability, tasks may be executed sequentially, in parallel, or synchronously.

The task set comprises 28 assembly operations with predefined precedence relationships, as reported in the original industrial case. Among these tasks, four operations (Tasks 5–8) are explicitly defined as collaborative tasks requiring simultaneous human–robot interaction. In these operations, the robot performs repetitive and physically demanding lifting of the upper module of the MBS, while the human operator guides and aligns the component onto the lower module. This synchronized manipulation reflects a typical HRC scenario aimed at reducing physical workload while maintaining assembly accuracy and operational safety.

Ergonomic risk is incorporated into the analysis using an EE-based approach. The EE values corresponding to human-performed tasks are directly adopted from Cai et al. [26], whereas the EE values for collaborative operations are computed according to the formulation presented in Section 5.1. The maximum allowable ergonomic load per station, denoted as ${EE}_{limit}$, is calculated based on Equation (32). This constraint ensures that the total physical workload assigned to each station remains within acceptable ergonomic limits, thereby integrating worker well-being into the decision-making process.

In addition to ergonomic considerations, all operational cost parameters, including station opening costs, human labor costs, robot operating costs, and robot energy consumption costs, are defined consistently with the parameter settings described in Section 5.1. This unified parameter structure enables a comprehensive evaluation of the trade-offs between economic efficiency and ergonomic performance. Detailed information on precedence relations, zoning constraints, task processing times, and ergonomic parameters is presented in the Supplementary Materials.

The proposed QLHH-GA is applied under these settings to evaluate its performance on the considered system. The results provide insights into the effectiveness of the approach in addressing practical HRC assembly challenges and demonstrate its potential to support decision-making in industrial environments.

Table 9 presents the performance of the proposed QLHH-GA under different robot configurations for the case study. The results show that increasing the number of robots leads to a reduction in cycle time, reflecting the enhanced processing capability provided by automation and human–robot collaboration. A similar trend is observed in total cost, which decreases substantially when transitioning from purely manual operations (Scenario 1) to hybrid configurations.

The minimum total cost is obtained in Scenario 3 with two robots, indicating that a moderate level of automation provides the most effective balance between operational cost and system performance. Although Scenario 4 yields a further reduction in the cycle time, the total cost increases compared to Scenario 3. This result reflects the diminishing economic returns associated with the additional robot due to higher investment and operating costs.

The variation in the observed maximum EE values across scenarios reveals a non-monotonic pattern. When the number of robots increases from 0 to 1, the maximum EE decreases, indicating a reduction in physical workload as a result of partial automation. In Scenario 3, where two robots are deployed, the maximum EE increases and approaches the allowable limit. This outcome indicates that human involvement becomes more intensive in collaborative tasks, leading to a higher localized workload despite automation. In Scenario 4, the maximum EE decreases again, implying that the additional robot contributes to a more balanced workload distribution across stations.

These results indicate that the relationship between automation level and ergonomic load is not linear. Instead, ergonomic outcomes depend on the interaction between task allocation decisions and the degree of HRC. The results demonstrate that certain levels of automation may increase localized workload due to intensified collaboration, even as overall system efficiency improves.

From a managerial perspective, the case study shows that the economic value of robot deployment in HRC assembly systems depends on the balance between cycle-time reduction and the additional cost of robotic resources. Although higher levels of robotization improve operational speed, the results show that the minimum total cost is achieved not in the most automated configuration but in an intermediate configuration with two robots. This finding indicates that increasing the level of automation does not always lead to proportional economic benefits under a cost-per-cycle objective. The results also confirm that ergonomic feasibility can be maintained across all scenarios when workload is explicitly incorporated into the decision process. In addition, the absence of a monotonic decrease in maximum EE values indicates that ergonomic performance is influenced by task allocation and process mode selection, rather than by automation intensity alone. For practitioners, these findings indicate that HRC system design should rely on integrated optimization of task assignment, process mode selection, and robot allocation, rather than on isolated investment decisions. In this context, the proposed approach provides a decision-support framework for identifying HRC configurations that balance productivity, cost efficiency, and worker well-being.

## 6. Conclusions

This study addresses the straight ergonomic mixed-model assembly line balancing problem under human–robot collaboration by developing an integrated mathematical modeling and computational framework. The proposed MILP model jointly considers task assignment, resource allocation, process mode selection, and scheduling decisions while incorporating ergonomic constraints based on EE limits. This formulation enables the direct enforcement of ergonomic feasibility within the optimization process and provides a structured representation of HRC assembly systems.

A QLHH-GA is proposed to address the computational complexity associated with large-scale problem instances. The proposed algorithm integrates population-based evolutionary search with adaptive learning and hyper-heuristic strategies, forming a hybrid nature-inspired framework characterized by two interacting adaptation mechanisms. Candidate solutions evolve through variation and selective retention at the population level, while reinforcement feedback modifies heuristic-selection behavior at the decision level. This interaction enables the search process to adapt its operator-selection policy according to the current search state and the outcomes of previous actions.

The computational experiments demonstrate that the proposed approach performs effectively across different problem sizes. The QLHH-GA achieves solution quality comparable to that of the MILP model for small-sized instances and provides competitive results for medium-sized problems, where exact methods become computationally demanding. All obtained solutions satisfy the predefined ergonomic constraints, indicating that the proposed framework maintains feasible workload distributions while minimizing operational cost.

The results further indicate that the total cost is influenced by the combined effect of cycle time settings and resource allocation decisions. Although increased robot availability enables more flexible task assignments and the use of alternative processing modes, the minimum cost is not necessarily achieved at the highest level of automation. Instead, intermediate configurations may provide more efficient trade-offs between resource utilization and cost components.

Ergonomic performance is primarily influenced by task allocation and process mode selection, indicating that workload distribution plays a key role in achieving balanced system designs. The results show that the proposed model successfully maintains ergonomic feasibility while allowing the exploration of alternative cost-efficient configurations.

The applicability of the proposed framework is demonstrated through a literature-based industrial case study, which confirms its ability to generate feasible and implementable assembly line configurations under practical constraints. The case study illustrates the interaction between system configuration, cost structure, and ergonomic considerations, supporting the relevance of the proposed approach for real production environments.

Future research can extend the proposed framework by incorporating stochastic processing times and uncertainty in ergonomic parameters, thereby enabling a more realistic representation of variability in production environments. The inclusion of dynamic human-related factors, such as fatigue, learning, and recovery processes, would further improve the modeling of human performance in collaborative systems. In addition, the proposed modeling and solution framework can be adapted to different assembly line configurations, including U-shaped, parallel, and multi-line systems, allowing its applicability to be evaluated under alternative production structures. Such extensions would provide additional insight into the interaction between system design and operational performance. The integration of optimization models with simulation-based approaches or digital twin environments can also support advanced decision-making in dynamic and data-driven production systems.

## Figures and Tables

**Table 1:** Summary of solution approaches for cost and ergonomics in HRC assembly line balancing.

Study	Objective Function	Ergonomic Risk Assessment Method(s)	Solution Approaches (*)
Samouei and Ashayeri [33]	Cost and cycle time		MILP
Dalle Mura and Dini [34]	Line cost, number of skilled workers, and energy load variance among workers	EE	GA
Weckenborg and Spengler [14]	Cost	EE	MILP
Yaphiar, Nugraha, and Ma’ruf [16]	Cost		MILP
Abdous, Delorme, and Battini [35]	Cost and workers’ fatigue	Workers’ fatigue model	MO-MINLP
Yifan et al. [36]	Cost		CE-GACEA
Nugraha et al. [37]	Cost		MILP
Li, Janardhanan, and Tang [17]	Cost and cycle time		MILP, MMBO
Dalle Mura and Dini [38]	Number of skilled workers, cost, energy load variance	EE	MILP, GA
Kökhan and Baykoç [18]	Cost and number of stations		MILP, SA
Shan et al. [12]	Cycle time and cost		MILP, NSGA-II
Weckenborg, Thies, and Spengler [39]	Cost and workers’ biomechanical load	EE	MILP
Dalle Mura and Dini [40]	Cost and workload	Noise exposure and EE	GA
Huang et al. [13]	Ergonomic risk, energy consumption, and cycle time	EE	MDABCSB
Nourmohammadi, Masood, and Ng [10]	Three models (number of stations, cycle time, cost)		MILP
Bekdemir and Taşan [25]	Cost	EE	MILP
Yin et al. [41]	Cycle time, ergonomic risk and cost	EE	IMOPSO
Yilmaz et al. [19]	Cost, workload, and energy	Predetermined Motion Energy System	MILP
Cai et al. [26]	Cycle time, workload variance, and assembly complexity	EE and RULA	IMBOA
Yilmaz et al. [20]	Cost and energy		MILP, NSGA-II
Kang and Lee [21]	Cycle time, collaboration cost, smoothness index, energy consumption, number of stations	Energy consumption	Preemptive fuzzy multiple objective programming NSGA-II
This Study	Cost	EE	MILP, QLHH-GA

* CE-GACEA: Cross-Entropy & Genetic Algorithm Co-Evolutionary Approach, GA: Genetic Algorithm, IMBOA: Improved Multi-Objective Migrating Birds Optimization Algorithm, IMOPSO: Improved Multi-Objective Particle Swarm Optimization Algorithm, MDABCSB: Multi-Objective Discrete Artificial Bee Colony with Specialist Bees, MILP: Mixed-Integer Linear Programming, MMBO: Multi-Objective Migrating Bird Optimization, MO-MINLP: Multi-Objective Mixed-Integer Nonlinear Programming, NSGA-II: Non-Dominated Sorting Genetic Algorithm, SA: Simulated Annealing.

**Table 2:** Notation of the proposed mathematical model.

Indices and Sets	
*i*, *j*	Index of tasks, $i,j\in \left\{1,2,\ldots ,NT\right\}$
*k*, *l*	Index of stations, $k,l\in \left\{1,2,\ldots ,NS\right\}$
*m*	Index of product models, $m\in \left\{1,2,\ldots ,NM\right\}$
*r*	Index of robots, $r\in \left\{1,2,\ldots ,NR\right\}$
*p*	Index of process alternatives, $p\in \left\{h,\sigma ,\tau \right\},$ where h is a human, σ is a robot, and τ is a collaboration of both
Parameters	
*c*	Cycle time
NT	Total number of assembly tasks
NS	Maximum number of stations
NM	Total number of product models
NR	Total number of robots
$t_{\mathrm{imp}}$	Processing time of task i of a product of model m by process alternative p
${\mathrm{EE}}_{\mathrm{imp}}$	Ergonomic risk of task i of model m by process alternative p
${\mathrm{cs}}_{k}$	Cost per unit time for opened station k
${\mathrm{ch}}_{k}$	Human labor cost per unit time for station k (applies only if a human operator is assigned to that station).
${\mathrm{cr}}_{r}$	Cost per unit time for robot r, reflecting the purchase cost and energy consumption during standby
${\mathrm{ce}}_{r}$	Cost of energy consumption per unit time for robot r during task execution.
${\mathrm{EE}}_{\mathrm{limit}}$	The maximum energy expenditure allowed per station
ψ	A sufficiently large positive number
$e_{\mathrm{ij}}$ = $\left\{\begin{array}{c} 1,\;if\;task\;i\;is\;an\;immediate\;predecessor\;of\;task\;j;\\ 0,\;otherwise \end{array}\right.$	
$\rho_{\mathrm{ij}}$ = $\left\{\begin{array}{c} 1,\;if\;task\;i\;and\;j\;must\;be\;allocated\;to\;the\;same\;station;\\ 0,\;otherwise \end{array}\right.$	
$\eta_{\mathrm{ij}}$ = $\left\{\begin{array}{c} 1,\;if\;task\;i\;and\;j\;cannot\;be\;allocated\;to\;the\;same\;station;\\ 0,\;otherwise \end{array}\right.$	
Decision variables	
$s_{\mathrm{im}}$	Start time of task i of model m in the station it is allocated to
$x_{\mathrm{impk}}$ = $\left\{\begin{array}{c} 1,\;if\;task\;i\;of\;model\;m\;is\;assigned\;to\;station\;k\;and\;processed\;by\;alternative\;p;\\ 0,\;otherwise \end{array}\;\;\;\;\;\;\;\;\;\;\right.$	
$z_{\mathrm{rk}}$ = $\left\{\begin{array}{c} 1,\;if\;robot\;r\;is\;allocated\;to\;station\;k;\\ 0,\;otherwise \end{array}\right.$	
${\mathrm{ws}}_{k}$ = $\left\{\begin{array}{c} 1,\;if\;station\;k\;is\;opened;\\ 0,\;otherwise \end{array}\right.$	
$h_{k}$ = $\left\{\begin{array}{c} 1,\;if\;a\;human\;is\;assigned\;to\;station\;k;\\ 0,\;otherwise \end{array}\right.$	
$w_{\mathrm{ijm}}$ = $\left\{\begin{array}{c} 1,\;if\;task\;i\;is\;scheduled\;before\;task\;j\;of\;model\;m;\\ 0,\;otherwise \end{array}\right.$	

**Table 3:** Main control parameters and their levels used in the Taguchi-based tuning of the algorithms.

Algorithm	Factors	Level 1	Level 2	Level 3
QLHH-GA	Population size (NP)	80	100	120
	Crossover probability ($P_{c}$)	0.6	0.7	0.8
	Initial exploration rate ($\varepsilon_{0}$)	0.10	0.20	0.30
GA	Population size (NP)	80	100	120
	Crossover probability ($P_{c}$)	0.6	0.7	0.8
	Mutation probability ($P_{m}$)	0.01	0.05	0.10
ABC	Food sources (NP)	80	100	120
	Abandonment limit (limit)	30	50	60
PSO	Swarm size (NP)	80	100	120
	Inertia weight (w)	0.75	0.80	0.90
	Acceleration coefficients ($c_{1}=c_{2}$)	1.5	2.0	2.5

**Table 4:** Performance comparison between MILP and QLHH-GA when τ = 100.

Instance	Size	Problem	Robots	Cycle Time (s)	MILP				QLHH-GA		
					Cost	Maximum EE	CPU (s)	Gap (%)	Cost	Maximum EE	RPD
1	Small-sized	P1 I = 11 K = 3 M = 1 ${EE}_{limit}$ = 15.49	0	16	100.8	15.3	3	1.00	100.8	13.7	0.00
2			1	14	91.5	12.8	4	0.01	92.4	12.5	0.93
3			2	12	81.4	10.0	13	0.09	81.9	14.3	0.53
4			3	10	71.4	12.6	15	0.06	71.8	8.1	0.51
5		P2 I = 11 K = 3 M = 1 ${EE}_{limit}$ = 13.84	0	65	390.6	12.7	2	1.00	409.5	13.2	4.84
6			1	55	342.7	10.7	2	1.00	363.3	12.4	6.01
7			2	48	313.7	9.6	2	0.03	332.5	9.2	6.02
8			3	45	293.3	12.2	2	0.10	319.5	9.8	8.94
9		P3 I = 19 K = 3 M = 3 ${EE}_{limit}$ = 20.25	0	130	819.0	20.2	2	0.10	819.0	20.2	0.00
10			1	120	772.2	18.2	1800	0.91	772.2	18.2	0.00
11			2	105	689.9	20.1	1800	0.82	689.9	20.1	0.00
12			3	95	652.1	14.7	1800	0.83	652.1	14.7	0.00
13	Medium-sized	P4 I = 29 K = 4 M = 1 ${EE}_{limit}$ = 20.30	0	85	714.0	20.0	1800	1.00	714.0	20.0	0.00
14			1	80	689.3	19.6	1800	0.84	686.5	18.4	−0.41
15			2	65	595.4	14.3	1800	0.86	602.0	14.3	1.11
16			4	60	558.4	13.2	1800	0.92	575.8	14.0	3.12
17		P5 I = 30 K = 6 M = 1 ${EE}_{limit}$ = 12.35	0	70	735.0	12.0	1800	1.00	735.0	12.0	0.00
18			2	65	680.6	11.1	1800	0.94	680.6	11.3	0.00
19			4	45	605.9	10.1	1800	0.80	611.9	11.3	0.99
20			6	42	580.2	7.8	1800	1.00	582.8	8.7	0.46
21		P6 I = 45 K = 6 M = 1 ${EE}_{limit}$ = 20.89	0	110	1386.0	20.7	1800	1.00	1386.0	20.7	0.00
22			2	95	1232.0	20.7	1800	1.00	1197.0	20.6	−2.84
23			4	85	1116.5	19.4	1800	0.90	1097.7	19.8	−1.69
24			6	75	1104.8	19.8	1800	1.00	1025.3	18.4	−7.19

**Table 5:** Performance comparison of QLHH-GA and benchmark metaheuristics when τ = 100.

Instance	Size	Problem	Robots	Cycle Time (s)	QLHH-GA			GA			PSO			ABC		
					Cost	Maximum EE	RPD	Cost	Maximum EE	RPD	Cost	Maximum EE	RPD	Cost	Maximum EE	RPD
1	Small-sized	P1 I = 11 K = 3 M = 1 ${EE}_{limit}$ = 15.49	0	16	100.8	13.7	0.00	100.8	14.9	0.00	100.8	14.9	0.00	100.8	13.7	0.00
2			1	14	92.4	12.5	0.00	93.5	12.5	1.23	93.5	12.5	1.23	92.4	12.5	0.00
3			2	12	81.9	14.3	0.00	83.8	14.3	2.42	83.8	14.3	2.42	81.9	14.3	0.00
4			3	10	71.8	8.1	0.00	73.4	6.2	2.26	73.4	6.2	2.26	71.8	8.1	0.00
5		P2 I = 11 K = 3 M = 1 ${EE}_{limit}$ = 13.84	0	65	409.5	13.2	0.00	409.5	13.2	0.00	409.5	13.2	0.00	409.5	13.2	0.00
6			1	55	363.3	12.4	0.00	367.6	12.4	1.18	367.6	12.4	1.18	363.3	12.4	0.00
7			2	48	332.5	9.2	0.00	337.5	8.4	1.50	337.5	8.4	1.50	334.1	9.2	0.46
8			3	45	319.5	9.8	0.00	319.5	9.8	0.00	328.4	7.7	2.78	321.4	9.8	0.60
9		P3 I = 19 K = 3 M = 3 ${EE}_{limit}$ = 20.25	0	130	819.0	20.2	0.00	819.0	20.2	0.00	819.0	20.2	0.00	819.0	20.2	0.00
10			1	120	772.2	18.2	0.00	794.4	20.2	2.88	792.4	20.2	2.62	785.3	18.2	1.70
11			2	105	689.9	20.1	0.00	745.7	18.2	8.09	711.8	18.2	3.18	720.9	18.2	4.49
12			3	95	652.1	14.7	0.00	677.9	9.2	3.96	696.8	11.4	6.85	677.9	9.2	3.96
13	Medium-sized	P4 I = 29 K = 4 M = 1 ${EE}_{limit}$ = 20.30	0	85	714.0	20.0	0.00	714.0	20.0	0.00	714.0	20.0	0.00	714.0	20.0	0.00
14			1	80	686.5	18.4	0.00	708.9	18.4	3.27	708.9	18.4	3.27	687.7	18.4	0.18
15			2	65	602.0	14.3	0.00	608.4	13.9	1.07	608.4	13.9	1.07	603.6	14.3	0.27
16			4	60	575.8	14.0	0.00	602.4	11.5	4.63	602.4	11.5	4.63	578.2	16.6	0.42
17		P5 I = 30 K = 6 M = 1 ${EE}_{limit}$ = 12.35	0	70	735.0	12.0	0.00	735.0	12.0	0.00	735.0	12.0	0.00	735.0	12.0	0.00
18			2	65	680.6	11.3	0.00	680.6	11.9	0.00	680.6	11.9	0.00	680.6	12.0	0.00
19			4	45	611.9	11.3	0.00	636.3	10.9	3.98	636.3	10.9	3.98	613.4	10.9	0.24
20			6	42	582.8	8.7	0.00	624.5	9.0	7.15	624.5	9.0	7.15	602.8	8.2	3.42
21		P6 I = 45 K = 6 M = 1 ${EE}_{limit}$ = 20.89	0	110	1386.0	20.7	0.00	1386.0	20.7	0.00	1386.0	20.7	0.00	1386.0	20.7	0.00
22			2	95	1197.0	20.6	0.00	1261.1	18.6	5.35	1261.1	18.6	5.35	1240.4	19.4	3.63
23			4	85	1097.7	19.8	0.00	1184.2	19.2	7.89	1184.2	19.2	7.89	1146.5	18.8	4.45
24			6	75	1025.3	18.4	0.00	1125.8	16.1	9.80	1125.8	16.1	9.80	1094.8	19.8	6.78
25	Large-sized	P7 I = 70 K = 8 M = 1 ${EE}_{limit}$ = 27.51	0	450	7560.0	26.6	0.00	7560.0	26.6	0.00	7560.0	26.6	0.00	7560.0	26.6	0.00
26			2	395	6919.3	25.5	0.00	7065.7	27.3	2.12	7065.7	27.3	2.12	7065.7	27.3	2.12
27			4	355	6527.8	23.0	0.00	6551.1	25.3	0.36	6576.0	25.3	0.74	6542.3	25.3	0.22
28			8	295	5918.0	25.1	0.00	5928.7	26.3	0.18	5971.7	18.5	0.91	5928.3	24.5	0.17
29		P8 I = 75 K = 7 M = 1 ${EE}_{limit}$ = 31.41	0	240	3528.0	30.0	0.00	3528.0	30.0	0.00	3528.0	30.0	0.00	3528.0	30.0	0.00
30			2	200	3014.1	22.4	0.00	3092.0	25.7	2.58	3092.0	25.7	2.58	3017.4	25.8	0.11
31			4	180	2830.7	24.2	0.00	2929.3	27.1	3.48	2929.3	27.1	3.48	2842.1	27.8	0.40
32			6	150	2567.2	18.3	0.00	2593.5	17.2	1.02	2593.5	17.2	1.02	2576.4	17.2	0.36
33		P9 I = 89 K = 6 M = 1 ${EE}_{limit}$ = 47.00	0	90	1134.0	46.3	0.00	1134.0	46.3	0.00	1134.0	46.3	0.00	1134.0	46.3	0.00
34			1	85	1071.0	44.4	0.00	1071.0	44.4	0.00	1096.0	44.4	2.33	1071.0	44.4	0.00
35			3	75	982.7	44.5	0.00	986.0	43.8	0.33	1027.0	37.1	4.51	1001.7	36.0	1.94
36			6	60	859.6	32.8	0.00	867.0	37.1	0.86	900.6	26.3	4.77	887.3	32.3	3.22

**Table 6:** Mean RPD performance of the algorithms across varying time limit parameters (τ).

Problem	QLHH-GA			GA			PSO			ABC		
	*τ* = 60	*τ* = 80	*τ* = 100	*τ* = 60	*τ* = 80	*τ* = 100	*τ* = 60	*τ* = 80	*τ* = 100	*τ* = 60	*τ* = 80	*τ* = 100
P1	0.00	0.00	0.00	1.48	1.48	1.48	1.48	1.48	1.48	0.00	0.00	0.00
P2	0.00	0.00	0.00	1.36	0.67	0.67	1.36	1.36	1.36	0.26	0.28	0.26
P3	0.00	0.00	0.00	3.73	3.73	3.73	3.16	3.16	3.16	2.54	2.54	2.54
P4	0.00	0.00	0.00	2.12	2.23	2.24	2.12	2.23	2.24	0.23	0.22	0.22
P5	0.00	0.00	0.00	2.77	2.76	2.78	2.77	2.76	2.78	0.70	1.01	0.92
P6	0.00	0.00	0.00	5.11	4.83	5.76	5.11	4.83	5.76	3.59	3.10	3.72
P7	0.00	0.00	0.00	0.91	0.92	0.66	0.91	0.92	0.94	0.62	0.62	0.63
P8	0.00	0.00	0.00	1.75	1.74	1.77	1.75	1.74	1.77	0.25	0.19	0.22
P9	0.00	0.00	0.00	0.34	0.08	0.30	2.61	2.71	2.90	1.08	1.05	1.29
Mean RPD	0.00	0.00	0.00	2.18	2.05	2.16	2.37	2.36	2.49	1.03	1.00	1.09

**Table 7:** Ablation comparison of random and Q-learning-based LLH selection.

Performance Measure		P7	P8	P9
Robots		8	6	6
Cycle time (s)		295	150	60
Random LLH selection	Cost (mean ± SD)	5932.282 ± 16.579	2581.017 ± 12.182	899.616 ± 23.751
	Best cost	5921.426	2569.994	866.974
	Maximum EE (mean ± SD)	24.320 ± 3.367	17.810 ± 0.712	29.644 ± 5.461
	Maximum EE (Best)	18.450	17.150	22.880
Q-learning-based LLH selection	Cost (mean ± SD)	5919.212 ± 1.445	2567.770 ± 0.793	866.191 ± 3.832
	Best cost	5917.965	2567.240	859.552
	Maximum EE (mean ± SD)	25.072 ± 1.959	18.408 ± 1.658	29.656 ± 2.177
	Maximum EE (Best)	21.880	16.910	27.240
Mean-cost improvement (%)		0.220	0.513	3.715

**Table 8:** Nemenyi post hoc test results.

	QLHH-GA	GA	PSO	ABC
QLHH-GA	1.000	<0.001	<0.001	0.082
GA	<0.001	1.000	0.844	0.058
PSO	<0.001	0.844	1.000	0.005
ABC	0.082	0.058	0.005	1.000

**Table 9:** Results of the case study under different robot configurations using the QLHH-GA.

Problem	Scenario	Number of Robots	Number of Stations	Number of Tasks	Cycle Time (s)	*EE_limit_*	Total Cost	Maximum EE
Case Study	Scenario 1	0	3	28	95	40.4	598.50	38.0
	Scenario 2	1	3	28	90	40.4	567.00	36.0
	Scenario 3	2	3	28	80	40.4	384.25	39.9
	Scenario 4	3	3	28	60	40.4	411.74	37.0

## Data Availability

The data supporting the findings of this study are available within the article and its Supplementary Materials.

## Supplementary Materials

The following supporting information can be downloaded at https://www.mdpi.com/article/10.3390/biomimetics11080600/s1, Table S1: Benchmark instances.

## Abbreviations

The following abbreviations are used in this manuscript:

ABC	Artificial Bee Colony
ALBP	Assembly Line Balancing Problem
EE	Energy Expenditure
Ergo-MMALBP-HRC	Ergonomic Mixed-Model Assembly Line Balancing Problem with Human–Robot Collaboration
GA	Genetic Algorithm
HRC	Human–Robot Collaboration
LLH	Low-Level Heuristic
MILP	Mixed-Integer Linear Programming
MMBO	Migrating Birds Optimization
NSGA-II	Non-Dominated Sorting Genetic Algorithm II
PSO	Particle Swarm Optimization
QLHH-GA	Q-Learning-Based Hyper-Heuristic Genetic Algorithm
RL	Reinforcement Learning
SA	Simulated Annealing
WMSDs	Work-Related Musculoskeletal Disorders

## Appendix A

**Table A1:** Parameter settings of the considered metaheuristics.

Algorithm	Parameter	Value	Setting Method
QLHH-GA	Population size (NP)	80	Taguchi (main)
	Crossover probability ($P_{c}$)	0.60	Taguchi (main)
	Initial exploration ($\varepsilon_{0}$)	0.30	Taguchi (main)
	Mutation probability ($P_{m}$)	0.01	Literature-based [55]
	Elite count ${(n}_{elite})$	0.29	Literature-based [55]
	Learning rate (λ)	0.10	Literature-based [56]
	Discount factor (γ)	0.80	Literature-based [56]
	Minimum exploration ($\varepsilon_{min}$)	0.05	Fixed
	Exploration decay (δ)	0.9999	Literature-based [49]
GA	Population size (NP)	120	Taguchi (main)
GA	Crossover probability ($P_{c}$)	0.60	Taguchi (main)
GA	Mutation probability ($P_{m}$)	0.05	Taguchi (main)
GA	Elite count ${(n}_{elite})$	0.29	Literature-based [55]
PSO	Swarm size (NP)	80	Taguchi (main)
PSO	Inertia weight (w)	0.90	Taguchi (main)
PSO	Cognitive coefficient $c_{1}$	2	Taguchi (main)
PSO	Social coefficient $c_{2}$	2	Taguchi (main)
ABC	Number of food sources (NP)	120	Taguchi (main)
ABC	Abandonment limit (limit)	60	Taguchi (main)
